# Host-Response Engineering in Regenerative Cardiology: Mechanistic Boundaries Between Remuscularization and Immune Reprogramming

**DOI:** 10.3390/biology15201884

**Published:** 2026-10-19

**Authors:** Luca Bonanni, Nicola Ferri

**Affiliations:** 1 Department of Medicine, Ospedale dell’Angelo, 30174 Venice, Italy; 2 Department of Medicine, University of Padua, 35122 Padova, Italy; nicola.ferri@unipd.it

**Keywords:** myocardial infarction, cardiac regeneration, host-response engineering, sterile inflammation, macrophages, efferocytosis, extracellular vesicles, mesenchymal stromal cells, biomaterials, remuscularization

## Abstract

Regenerative cardiology has bifurcated into true remuscularization and host-response engineering. The first uses pluripotent-stem-cell-derived cardiomyocytes or engineered heart muscle to achieve durable contractile replacement; the second comprises adult stromal-cell products, extracellular vesicle preparations and biomaterials that may redirect endogenous repair without generating meaningful myocardium. This mechanistic narrative review reconstructs post-ischemic sterile inflammation as a temporally and spatially organized program and evaluates whether these platforms converge on shared causal pathways. Evidence is classified claim by claim using separate descriptive categories for therapeutic efficacy and mechanistic causal support. The platforms diverge proximally through administered-cell death and clearance, vesicle biodistribution and cargo transfer, or material–tissue interactions. Their most defensible distal convergence is incomplete and centers on monocyte/macrophage balance, efferocytosis, immune–fibroblast signaling and the macrophage–fibroblast–extracellular-matrix axis. Causal support is strong for selected mediators and preparations but cannot be generalized across product classes. No cross-platform convergence on therapeutically meaningful cardiomyocyte generation has been demonstrated. Human studies show limited target-engagement signals without evidence that they localize to myocardium or mediate benefit. Route, timing, disease stage and product identity should therefore be treated as mechanistic variables in preclinical design and clinical translation.

## Simple Summary

Cardiac regenerative medicine began with the idea that injected cells would replace heart muscle lost after myocardial infarction. Genetic tracking later showed that most adult cell products neither persist nor generate meaningful numbers of new cardiomyocytes, although some studies still reported modest functional benefit. The field has therefore divided into two branches. Pluripotent-stem-cell-derived cardiomyocytes and engineered heart muscle aim to create durable contractile tissue. Adult stromal cells, extracellular vesicle preparations and biomaterials are more plausibly understood as modifiers of the patient’s own repair response. This response is driven by a timed sequence of immune and stromal events that remove dead tissue, control inflammation, rebuild vessels and organize the scar. We examine whether different regenerative platforms converge on this endogenous program, particularly on communication among macrophages, fibroblasts and the extracellular matrix. The evidence supports some causal links but does not establish a single mechanism shared by all products. Timing, route of delivery and product identity are therefore part of the mechanism rather than practical details. We also identify the experiments and patient measurements needed to distinguish genuine immune reprogramming from association.

## 1. Introduction

The intellectual architecture of adult cardiac regenerative medicine was established early and on premises that later proved untenable. In 2001, lineage-negative c-kit-positive bone marrow cells injected into the infarct border were reported to regenerate 68% of the infarcted ventricle within nine days [1]. The claim was arithmetically spectacular and conceptually simple: cardiomyocytes are lost, therefore cardiomyocytes must be supplied. Within three years, two groups using genetic fate mapping and highly purified hematopoietic stem cells found no transdifferentiation; the transplanted cells adopted mature hematopoietic fates in the ischemic myocardium and largely disappeared [2,3]. A decade later, inducible *Kit* lineage tracing placed the contribution of endogenous c-kit-expressing cells to the cardiomyocyte pool at approximately 0.03% or less, with the same lineage contributing substantially to endothelium [4]. Radiocarbon dating of human cardiomyocyte nuclei had already fixed the ceiling on lifetime renewal at roughly 1% annually at age 20 and 0.3% at age 75, so that fewer than half of cardiomyocytes are exchanged across a normal lifespan [5]. A consensus statement drew the inference that adult mammalian cardiomyogenesis is real but quantitatively trivial relative to the tens of grams of muscle lost in a large infarction [6].

It would be convenient, and wrong, to conclude that the replacement paradigm has failed. It has failed for a specific class of products. Pluripotent-stem-cell-derived cardiomyocytes form large, vascularized, electromechanically coupled grafts in non-human primates [7,8], and engineered heart muscle allografts implanted epicardially in macaques showed sustained retention, with a single first-in-human observation of graft persistence in one trial participant [9]. Regenerative cardiology has not undergone a single linear transition; it has bifurcated into two mechanistic families that coexist. The first, remuscularization, delivers contractile cells intended to persist, couple and substitute for lost muscle. The second, which we will call host-response engineering, delivers adult stromal-cell products, extracellular vesicles or biomaterials that are not intended to become myocardium and, as a matter of demonstrated fact, do not. This review concerns the second family. The first appears only as a boundary condition, because it establishes that tissue replacement is biologically attainable and therefore forces the argument about adult products to be made on its own evidence rather than by default.

Within the host-response branch an awkward residue of efficacy survived the collapse of the replacement premise. Intramyocardial delivery of adult cell products produced small but reproducible improvements in ejection fraction and infarct geometry across many rodent studies. Either that benefit is an artifact of publication bias and underpowered design, or it is real and its mechanism was never the donor cell. The most informative experiment bearing on the question showed that intracardiac injection of two distinct progenitor populations, of the same cells killed by freeze–thaw, or of zymosan A—a fungal cell-wall preparation containing no cells at all—each produced comparable functional improvement after ischemia–reperfusion injury, each accompanied by regional accumulation of CCR2+ and CX3CR1+ macrophages, and none accompanied by new cardiomyocytes [10]. Saline injection produced a mild inflammatory response of its own without improving function, so the effect is not needle trauma, and the benefit persisted for at least eight weeks. Benefit tracked the host response, not the graft.

### 1.1. Operational Vocabulary

The field has used its central nouns loosely, and the argument cannot be made without fixing them. True myocardial regeneration means durable generation, maturation, vascularization, electrical integration and mechanical contribution of a quantitatively meaningful mass of new cardiomyocytes. Cardioprotection means reduction in initial cardiomyocyte injury or death, an event largely confined to the first hours. Tissue repair means restoration of structural integrity through debris clearance, vascular support, matrix organization, scar formation and limitation of adverse remodeling, and does not require new myocardium. Reverse remodeling means improvement in ventricular geometry or function, which is a chamber-level, load-dependent measurement and is not evidence of cardiomyogenesis. Immune reprogramming, the organizing concept of this review, means a therapeutically induced, temporally and spatially appropriate redirection of immune-cell states and intercellular signaling that changes the subsequent trajectory of tissue repair. The definition excludes generalized immunosuppression, reduced leukocyte counts, and movement along the M1/M2 axis, a vocabulary whose inadequacy as a description of in vivo macrophage biology has been formalized twice over [11,12].

Six further terms are kept strictly separate throughout, because the literature routinely slides between them. Immune modulation means that an immune parameter changed. Immune dependence means that removing an immune population abolished the effect. Immune mediation means that the immune change lies on the causal path between treatment and outcome, which requires temporal ordering and a mediation model, not merely a depletion experiment. Structural repair, functional improvement and clinical efficacy are three distinct outcome domains. Demonstrating any one of them says nothing automatic about the others.

### 1.2. What This Review Adds

Existing reviews have covered post-infarction inflammation, the stromal cell secretome and vesicle cargo separately. What has not been attempted is a disciplined cross-platform test of convergence in which the type and directness of evidence for each claimed shared mechanism are stated explicitly and claim by claim. We therefore ask: across adult cell products, extracellular vesicle preparations and biomaterials used after ischemic cardiac injury, what evidence shows that benefit is mediated by a temporally and spatially controlled redirection of the host immune–stromal repair response rather than by cardiomyocyte replacement, and where does that interpretation remain incomplete or contradicted?

The answer we defend has a specific shape, and it is a working model rather than a settled classification. The bifurcation into two mechanistic families is a device we propose for reading the evidence, not an established taxonomy of the field, and the boundary between the branches is permeable. Within that frame the platforms diverge proximally and may converge distally, incompletely and to different degrees, on the macrophage–fibroblast–extracellular-matrix axis. No cross-platform convergence on therapeutically meaningful cardiomyocyte generation has been demonstrated. Convergence does not make the platforms interchangeable, does not establish that every observed benefit is immunologically mediated, and depends on timing, anatomical compartment, route, dose and injury context. It is far better established experimentally than clinically.

## 2. Literature Selection and Evidence Framework

### 2.1. Search

This is a mechanistic narrative review. It does not follow systematic-review methodology: there was no protocol registration, no prespecified inclusion algorithm, no duplicate screening and no risk-of-bias instrument, and no pooled estimates are reported. Study selection was purposive, prioritizing primary studies employing causal perturbation, human tissue studies and randomized trials.

The candidate literature was identified through targeted topical discovery searches and citation chaining from the reference lists of retrieved primary studies and a small number of orienting reviews. This is a structured narrative review. It is not a systematic review, a meta-analysis, a validated evidence-grading exercise or a formal risk-of-bias assessment, and it should not be read as one. The process was purposive rather than systematic: no structured Boolean strategy or formal screening log was used, the number of records screened was not tracked, and exhaustive coverage is not claimed. Selection bias cannot be excluded and was mitigated only by three deliberate practices: studies were sought for their decisiveness rather than their direction, negative and null results were retained wherever they bore on a graded claim, and every study carrying a graded claim was read in full text rather than from an abstract. Every study supporting a decisive claim was checked individually in PubMed and against the publisher record or DOI registry. For all studies retained in the claim-level evidence tables, the full text was examined during final verification. Two structural biases in this literature cannot be corrected by careful reading and are therefore declared rather than adjusted for. Preferential publication of positive preclinical experiments may overstate the reproducible effect, while negative perturbation experiments may be underrepresented; the extent of either bias cannot be estimated from this purposive review. Selective reporting within studies compounds this, as does the heterogeneity of animal models, therapeutic products, doses, delivery routes, treatment timing and endpoints, which makes the effects tabulated here not straightforwardly comparable across rows. No formal publication-bias testing was performed, and the purposive design of this review would not support one.

The targeted search was last checked on 8 August 2026; two outstanding full texts were examined during the September 2026 revision. Indexing of the most recent months is incomplete in any interface and no claim of exhaustive coverage of 2026 is made; the evidence base extends reliably through late 2025, with individually verified 2026 additions. Search concepts combined the replacement premise and its refutation, the post-ischemic immune program, the three therapeutic platforms, remuscularization as a boundary condition, and human translation including named trials and anti-inflammatory interventions. Reviews were used for terminology and citation chaining and are not substituted for primary evidence anywhere in the argument.

### 2.2. A Two-Axis Claim-Level Descriptive Framework

The E/C framework is an author-developed, operational, descriptive, claim-level taxonomy for structured mechanistic comparison, applied to claims rather than to studies. It is not a validated score, a consensus instrument, a formal hierarchy of evidence, a risk-of-bias tool, a clinical decision rule or a quantitative grading system. It separates evidence of therapeutic efficacy from evidence of mechanistic causality. The labels are not combined into a composite score, used to determine study eligibility or used for quantitative comparisons between studies. This distinction matters because a randomized trial can provide strong evidence for therapeutic efficacy while providing none for the immune mechanism proposed to explain it, whereas a perturbation experiment in a mouse can establish immune necessity without establishing clinical benefit. For each decisive study we therefore ask seven separate questions, and a study may answer some of them while leaving others untouched.

The practical purpose of asking them separately is to make three habitual errors visible before they are committed. The first is reading a therapeutic-efficacy result as mechanistic support, which the E and C axes prevent by construction. The second is treating immune target engagement as causal mediation, which the separation of questions 2 and 5 exposes. The third is comparing platforms as though evidence obtained for one licensed a claim about another, which the context tags and the per-claim rows make explicit. Used this way, the taxonomy identifies which causal link in a proposed mechanism remains untested, and so indicates which experiment, biomarker, localization method or clinical endpoint would actually advance the claim rather than decorate it. Its most concrete use is negative: an improvement in ventricular function is evidence of neither remuscularization nor immune mediation, and the framework makes the gap between the observation and either interpretation countable. None of this makes the taxonomy a validated instrument, and it has not been externally tested or adopted by consensus.

1. Therapeutic efficacy—did the intervention change a structural, functional or clinical outcome relative to an adequate comparator? This question alone determines the E label.
2. Immune target engagement—was the proposed immune target measurably modified by biomarker, cell-state or transcriptional readout? Engagement without a downstream outcome supports C1 and no more.
3. Necessity—was the proposed mechanism removed, blocked or deleted, and was the benefit lost? Selective removal supports C3 for the link tested; non-selective removal supports C2.
4. Sufficiency—was the proposed mechanism supplied in isolation, and was the benefit reproduced? Sufficiency and necessity are graded separately and are never inferred from one another.
5. Mediation—was the measured engagement shown to lie on the causal path to the measured outcome? Randomization to treatment does not establish this, and in the absence of a mediation analysis the label is C0.
6. Cardiac localization—was the mechanism demonstrated in myocardium, as opposed to blood, lung, lymph node or another compartment? An extracardiac demonstration is recorded with a cross-organ tag and does not license a cardiac claim.
7. Clinical relevance—does the model, route, timing and population correspond to the human setting in which the therapy would be used? This governs the context tag and the interpretation, not the grade itself.

The E axis classifies the evidence supporting a therapeutic-efficacy claim. E–: not applicable because the claim does not concern therapeutic efficacy. E0: efficacy is relevant to the claim but was not tested or cannot be inferred. E1: an exploratory, uncontrolled or associative signal. E2: controlled evidence limited by a single study, model, small sample or surrogate outcome. E3: high-confidence evidence for the efficacy claim, such as an adequately controlled randomized trial or a robust meta-analysis, interpreted only within the population and endpoint actually studied.

The C axis classifies the evidence supporting a proposed causal mechanism. C0: the mechanism was not measured or tested. C1: mechanistic association, biomarker change, spatial co-localization, temporal correspondence or target engagement without causal perturbation. C2: causal perturbation supports pathway dependence, but the chain remains incomplete or alternative explanations such as toxicity, altered biodistribution or broader cellular depletion remain plausible. C3: direct evidence of necessity and/or sufficiency for the precisely stated mechanistic claim, with appropriate controls. A C3 label applies only to the link that was tested and never licenses describing a whole pathway as established.

A non-applicable designation is required because several of the most important claims in this field are not mechanistic claims at all. C–: not applicable, because the claim is descriptive, quantitative, histological, lineage-tracing-based or refutational rather than a proposed causal mechanism. C– does not indicate weak evidence. Lineage tracing that detects no new cardiomyocytes, radiocarbon dating that quantifies turnover, histology that shows a graft is present and vascularized, and fate mapping that refutes transdifferentiation are all direct, often decisive observations; they simply do not test necessity or sufficiency of a mechanism, and labeling them C3 would misrepresent them as mechanistic proof. The strength, directness and limitations of such observations are recorded in the experimental-test and limitation fields of Tables S1 and S2 instead. Conversely, C3 is never used to mean a high-confidence observation, strong negative evidence, absence of detection or a successful histological demonstration.

A separate context tag records where the evidence was obtained: H human, LA large animal, SA small animal, IV in vitro or ex vivo. The context tag is not a quality ranking. Human evidence is not automatically stronger mechanistically than animal perturbation evidence, and animal evidence is not evidence of human efficacy. Different mechanistic claims from one study receive different C labels, and where a study supports several distinct claims it occupies more than one row of Tables S1 and S2. DREAM-HF, for example, is E3-H for the absence of benefit on its primary endpoint and C0-H for immune mediation [13]; de Couto and colleagues provide E2-SA for therapeutic benefit, C2-SA for loss of benefit after broad myeloid depletion and C3-SA for sufficiency of transferred conditioned macrophages, without upgrading the untested downstream molecular steps [14]; and the lineage-tracing observation that no new cardiomyocytes were detected is C– rather than C3, because it is a direct observation and not a test of mechanism [10]. Ranges such as C1–C2 are not used: where uncertainty reflects two different claims, the claims are separated and classified individually. Tables S1 and S2 record each label against the exact claim it supports.

Three levels of evidence are kept apart throughout, and the distinction governs how every claim in this review is worded. The first is endogenous immune or stromal biology after cardiac injury: what the host does on its own. The second is evidence that a therapeutic intervention changes that biology. The third is evidence that the change causally mediates the therapeutic benefit. Evidence at the first level, however decisive, never establishes the second or the third; a knockout that worsens infarct healing says nothing about whether a stromal-cell product engages the same pathway. Nor is mediation inferred from temporal association, colocalization, transcriptional change, immune-cell abundance or the loss of statistical significance in a subgroup. Where only the first level is available, the text says so.

## 3. Two Branches of Regenerative Cardiology

### 3.1. Erosion of the Replacement Premise for Adult Cell Products

The replacement hypothesis was structurally fragile because it rested on cell-identity assignments made by immunofluorescence in tissue crowded with autofluorescent and overlapping cells. Genetic labeling removed the ambiguity. Cre-mediated marking of hematopoietic stem cells and their progeny showed that transplanted cells in infarcted myocardium generated granulocytes and monocytes, and purified c-*kit*-positive hematopoietic stem cells produced no detectable myocyte progeny [2,3]. Applied to endogenous *Kit*-expressing cells, inducible fate mapping returned a cardiomyocyte contribution two orders of magnitude below any plausible functional threshold [4]. The evidentiary status of the opposing literature was further weakened when an institutional review concluded that data from the laboratory that generated much of the original c-*kit* work were unreliable, leading to retraction of the first-in-human SCIPIO report [15].

Persistence data compounded the problem. Intravenously delivered mesenchymal stromal cells embolize almost quantitatively in the pulmonary microvasculature and disappear within roughly 24 h without reaching the heart in meaningful numbers [16]. Even for products delivered directly into myocardium, retention is measured in days. A therapy whose active agent is gone before granulation tissue has formed cannot be acting as replacement tissue, and this pharmacokinetic observation is arguably the most robust single argument against the replacement model for adult products.

### 3.2. Remuscularization as a Boundary Condition

The opposite result has been obtained where the product actually contains cardiomyocytes. Approximately one billion human embryonic stem cell-derived cardiomyocytes injected into infarcted macaque myocardium produced large, vascularized grafts with host–graft junctions and calcium transients synchronized to the host, together with ventricular arrhythmias [7]. A subsequent study using approximately 750 million cryopreserved cells reported durable grafts, electromechanical junctions and improved global left ventricular ejection fraction relative to controls, again with graft-associated arrhythmias in a subset [8]. More recently, epicardial engineered heart muscle allografts were implanted in rhesus macaques at doses of 40 million cells per patch (1 × EHM) up to 200 million (5 × EHM, the maximum feasible dose), with sustained retention to six months and no arrhythmia or tumor formation across the 20 implanted animals, which together received 66 patches constructed from 2.64 × 10^9^ cells. In the randomized chronic heart-failure cohort seven macaques were assigned to engineered heart muscle, three to 2 × EHM and four to 5 × EHM, against seven controls. One animal in the 5 × EHM group died in the post-anesthesia recovery phase from low cardiac output syndrome, leaving six evaluable animals, three per dose. The grafts produced dose-dependent augmentation of target wall thickness (+1.5 ± 0.8 mm with 2 × EHM and +2.2 ± 0.1 mm with 5 × EHM, *n* = 3 per group, versus −0.03 ± 0.07 mm in seven controls), sustained improvement in wall thickening fraction in three of the six evaluable treated animals, and improved ejection fraction in three animals (+7 ± 3% versus −2 ± 2% in controls) [9]. The randomized comparison therefore provides a preliminary functional efficacy signal in a very small cohort, with improvement confined to a subset. The same product has now reached a clinical cohort. In an open-label, uncontrolled phase 1–2 study, twenty patients with heart failure and an ejection fraction of 35% or less received epicardial allografts of engineered heart muscle under immunosuppression; at the prespecified three-month interim analysis of the sixteen who received the maximal tolerated dose, the twelve evaluable patients showed a least-squares mean increase in target wall thickness of 4.5 mm, a 3.9-percentage-point rise in ejection fraction and a 6.7-point rise in the Kansas City Cardiomyopathy Questionnaire overall summary score [17]. Three qualifications belong with those numbers. There was no control group, the significance threshold was prespecified at *p* < 0.10 with 90% confidence intervals, and the quality-of-life result was *p* = 0.06, within the prespecified threshold, but remains difficult to interpret without a comparator. Every patient had at least one adverse event, 196 events occurred including 57 serious events, and three patients died, all deaths adjudicated as unrelated to the graft. The histological demonstration that the graft becomes vascularized human myocardium still rests on the single explanted heart reported earlier [9], not on this cohort.

The same report supplies the experiment that makes the bifurcation empirically testable rather than merely conceptual. In the authors’ own xenograft work in nude rats, viable and lethally irradiated engineered heart muscle produced similarly improved outcomes with notable macrophage infiltration, a muscularization-independent result of exactly the kind described in Section 3.3. In immunosuppressed allograft settings, by contrast, cardiomyocyte-containing grafts outperformed non-myocyte and non-viable grafts, which is what licenses the interpretation of functional remuscularization [9]. The viable-versus-non-viable comparison at matched geometry is thus the empirical discriminator between the two branches, and it is the same design that the Falsification Section proposes as falsifier F9 for biomaterials.

The same report includes a human observation that requires exact statement, because it is the single most over-interpreted result in this literature. The direct human histological evidence derives from a single participant in the BioVAT-HF-DZHK20 Phase 1/2 first-in-human trial, treated in a bridge-to-transplant scenario with epicardial engineered heart muscle allografts constructed from 400 million cardiomyocytes and stromal cells. Three months after implantation the patient underwent heart transplantation, permitting histological examination of the explanted heart. Grafted cardiomyocytes were retained and the graft was vascularized, although at a capillary density roughly fivefold lower than the recipient myocardium (187 ± 5 versus 963 ± 12 per mm^2^); the grafted cells remained markedly smaller and less mature than host cardiomyocytes; and although adherens-junction protein was detected, connexin 43 expression was sparse [9]. The observation therefore establishes cardiomyocyte retention and partial vascularization. It does not establish electromechanical integration, quantitative contractile contribution, clinical efficacy, generalizable safety or reproducibility. This histological inference pertains only to the explanted heart; the subsequently reported cohort outcomes [17] are uncontrolled clinical observations and provide no additional individual tissue evidence.

Two consequences follow for the argument. Remuscularization is possible, so the failure of adult products to remuscularize is a fact about those products rather than about the heart. And because remuscularization requires implanted contractile cells that persist, an intervention whose active agent is undetectable within a week cannot be explained that way. The bifurcation is therefore not a rhetorical device; it is what makes the host-response argument specific rather than merely negative [18,19]. Human embryonic stem cell-derived cardiovascular progenitors delivered in a fibrin patch, by contrast, sit awkwardly between the branches: the first-in-human study demonstrated feasibility and short-term safety without demonstrating engraftment, and the authors attributed any effect to paracrine action [20].

### 3.3. The Repair Paradox in the Host-Response Branch

Four lines of evidence converge on the proposition that, for adult products, the operative agent is the host. Clodronate depletion of macrophages, applied either four hours before or three days after infarction, attenuated rather than abolished the favorable effect of stromal-cell therapy on remodeling and function, and raised 30-day mortality in treated and untreated animals alike, with the deleterious effect partly reversed by macrophage restoration; the authors conclude that some, not all, of the protective effect is macrophage-mediated [21]. Substituting freeze–thaw-killed cells or a non-cellular innate stimulus reproduced both the macrophage accumulation and the functional benefit, and the same study abolished that benefit by two independent immune manipulations, broad immunosuppression with cyclosporine A and macrophage depletion with clodronate liposomes, while mice lacking CCR2 or CX3CR1 derived no benefit from cell therapy at all [10]. And in cardiosphere-derived cell therapy given shortly after reperfusion, macrophage depletion abolished cardioprotection while adoptive transfer of macrophages conditioned by the cells reproduced it and reduced cardiomyocyte death—necessity and sufficiency in the same cardiac model [14]. That last experiment is the strongest complete demonstration available for any adult cell product and anchors the remainder of this review.

The same logic now extends into the remuscularization branch, which complicates any attempt to keep the two families hermetically separate. Two perturbations in the same study make the point, and they are not equivalent. In wild-type recipients, transplanted neonatal cardiomyocytes improved ejection fraction and lateral wall thickness. In *Rag2*-deficient recipients, which lack mature T and B cells but retain myeloid and natural killer compartments, the same graft produced no functional improvement, ejection fraction and wall thickness declined slightly, and the graft was detected in one of seven hearts rather than three of seven. Pharmacological CCR2 inhibition in wild-type recipients removed the ejection-fraction and wall-thickness benefit, yet circumferential strain still improved, so regional contractility and pump function were dissociated rather than lost together [22]. Lymphocyte deficiency and selective CCR2 blockade are therefore different manipulations with different readouts, and neither should be described as abolishing the functional effect outright. In both arms the functional result was decoupled from graft survival, which the authors themselves note. Host immunity is thus a determinant of graft-based therapies as well as the mechanism of graft-free ones, though for different reasons.

Three cautions constrain how far this can be pushed. “No new cardiomyocytes” is a statement about detection thresholds; low-level cardiomyogenesis exists and is measurable in humans [5], and the therapeutic question is whether it can be amplified. Improvement in ejection fraction, infarct size, viable mass, fibrotic fraction or capillary density is not evidence of regeneration under any circumstance, and that discipline must be applied to immune-mediated benefit exactly as it was applied to the replacement claims [6,23]. And the specificity of the Vagnozzi result to local injection, young animals and a defined delayed timing limits generalization to all products and all routes. That limitation is not hypothetical: the same laboratory subsequently randomized mice to receive 150,000 bone marrow mononuclear cells or saline into the border zone of an established chronic scar, eight weeks after ischemia–reperfusion injury, and found no improvement in fractional shortening, end-systolic volume, hypertrophy or border-zone fibrosis, whereas the concurrent one-week cohort improved [24]. The finding is specific to bone marrow mononuclear cells, the only product tested. Its most instructive feature is a dissociation: the cells still induced local inflammation in the chronic scar, with greater activated macrophage content than saline three days after injection, and produced no functional benefit. Inducing local inflammation was therefore not sufficient in this chronic-scar setting; the findings support, but do not prove, a role for disease stage or myocardial substrate in determining treatment response. Route and timing are developed in Section 7.3.

What is established at this point is narrow and defensible: for several adult cell products delivered into ischemically injured myocardium, functional benefit occurs without meaningful cardiomyocyte replacement, is abolished by myeloid depletion, and can be reproduced by non-cellular sterile stimuli or by transfer of conditioned macrophages. What remains inferred is that this generalizes across platforms. Strengthening causal confidence would require the same perturbation applied to different platforms in one model, which has not been done.

## 4. Sterile Inflammation as a Time-Dependent Repair Program

**Figure 1:**
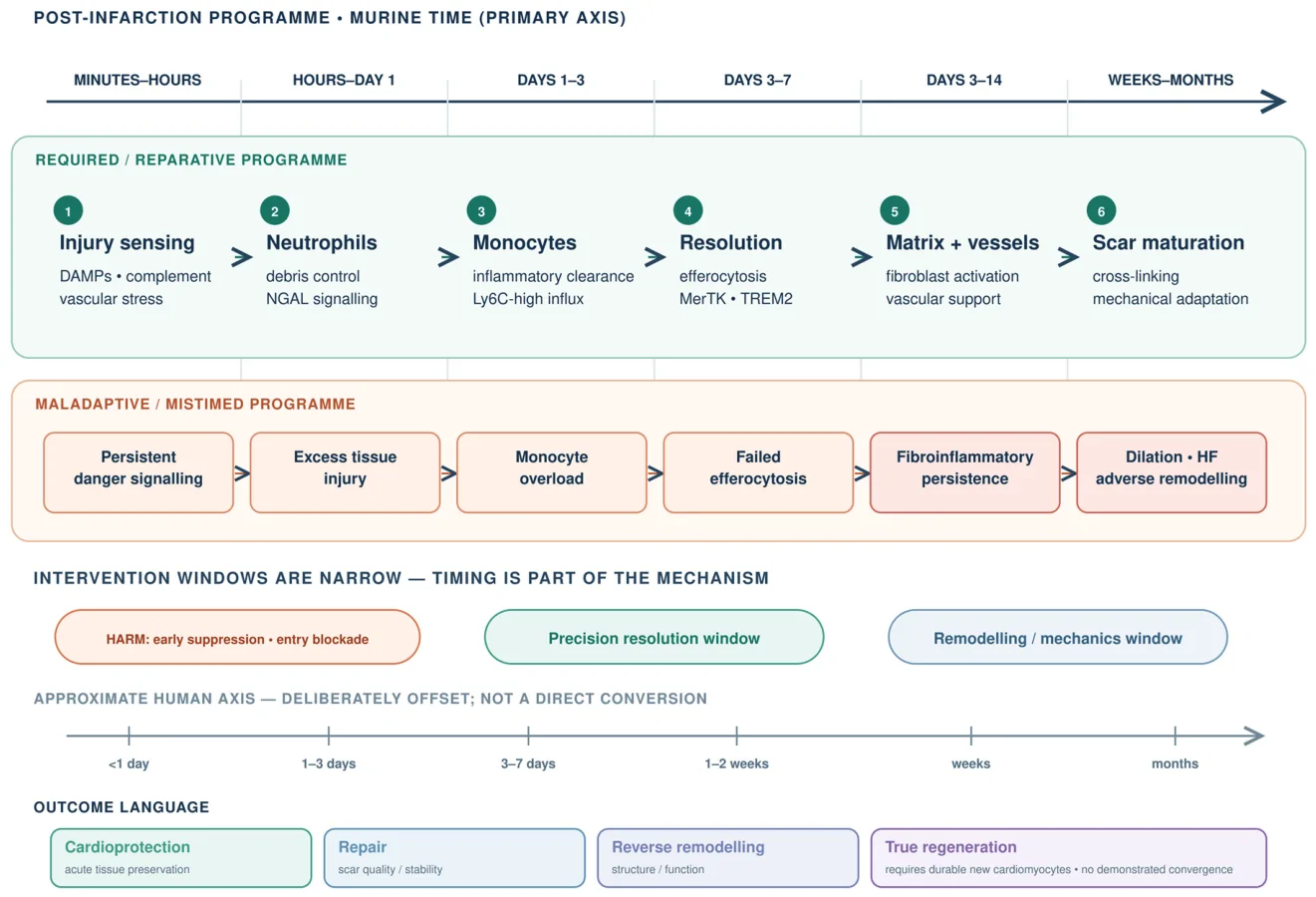
The post-infarction immune–repair clock. The primary axis shows an approximate murine sequence from injury sensing and neutrophil responses through monocyte recruitment, efferocytosis, matrix and vascular repair, and scar maturation. The separate human axis is deliberately offset and does not imply direct interspecies conversion. Parallel bands distinguish reparative responses from maladaptive or mistimed responses. Windows beneath them show the distinct timing of early suppression risk, resolution and later remodeling. Outcome labels distinguish acute cardioprotection, repair, reverse remodeling and true regeneration; durable new cardiomyocytes have not been demonstrated across the host-response platforms. Phase boundaries and intervention windows are schematic and depend on the injury model and treatment.

Any argument that therapies act by redirecting inflammation must first establish what the undisturbed program does and why interfering with it is dangerous as often as it is useful. Post-infarction inflammation is not a pathological accident superimposed on injury; it is the mechanism by which necrotic myocardium is removed and replaced by a mechanically competent scar [25]. The clinical history of the field is largely a record of learning this the hard way. Corticosteroids and nonsteroidal anti-inflammatory drugs given early after experimental infarction produce scar thinning and infarct expansion rather than protection [26,27]. The sequence summarized below, together with the microvascular component of reperfusion injury that runs in parallel with it, has been reviewed in detail elsewhere and is reconstructed here only to the depth the present argument requires [25,28]. Timing is therefore part of the mechanism, not a dosing detail, and any framework equating less inflammation with better healing is contradicted by data four decades old. This temporal architecture is summarized in Figure 1.

### 4.1. Minutes to Hours: Spatially Restricted Injury Sensing

Ischemic cardiomyocyte death releases nucleic acids, HMGB1, ATP and mitochondrial constituents that engage pattern-recognition receptors. Two cytosolic nucleic-acid-sensing pathways have been interrogated with genetic tools and are unusually well supported. Mice lacking cGAS, STING, IRF3 or the type I interferon receptor showed improved survival and reduced adverse remodeling after infarction, and antibody blockade of IFNAR reproduced the effect [29]; macrophage-restricted loss of cGAS shifted macrophage state and improved outcome in a parallel study [30]. The anatomical origin of the signal was subsequently localized: colonies of interferon-induced cells decorate the infarct border zone, and cardiomyocyte-selective deletion of *Irf3* abolished them, whereas deletion in fibroblasts, macrophages, neutrophils or endothelial cells did not [31]. Mechanically stressed border-zone cardiomyocytes with ruptured nuclei are the initiating cells. This converts type I interferon signaling from a diffuse inflammatory variable into a spatially defined target, and it is the clearest existing demonstration that the word “spatially” in our operational definition refers to something measurable. The NLRP3 inflammasome and downstream IL-1 constitute a second, partly independent amplifier [32].

### 4.2. The Neutrophil Phase

Neutrophils dominate the first day and are conventionally cast as agents of collateral damage. Depletion experiments do not support that reading: antibody-mediated neutrophil depletion worsened cardiac function, increased fibrosis and led to progressive heart failure, and did so by impairing acquisition of a reparative macrophage phenotype [33]. The mediator was identified and tested in both directions: neutrophil gelatinase-associated lipocalin was found in the neutrophil secretome, immunodepletion of it from that secretome blunted the effect, and administration of either the secretome or recombinant lipocalin restored the cardiac macrophage phenotype in depleted mice. Depletion also reduced macrophage MerTK expression and left more apoptotic cells within the infarct, so the neutrophil checkpoint and the efferocytosis checkpoint of Section 4.4 are mechanistically linked rather than parallel. Neutrophils simultaneously drive a systemic amplification loop through S100A8/A9 acting on marrow progenitors, and interrupting that loop improved outcome [34]. A second, independent line of evidence has since reinforced the same principle from an unexpected anatomical direction: spatial multiomic mapping of the acute infarct identified leukocyte entry through the endocardium and von Willebrand factor as an upregulated mediator, and functional blockade of von Willebrand factor reduced CCR2-positive monocyte infiltration and worsened cardiac function [35]. Two mechanistically unrelated blockades of early recruitment, in different laboratories, both produced harm. The therapeutic objective cannot be elimination of early recruitment.

### 4.3. Monocyte Recruitment and Macrophage Expansion

Monocyte recruitment follows a biphasic pattern, with Ly6C-high cells dominating days one to four and Ly6C-low cells thereafter, each phase associated with distinct programs [36]; the nuclear receptor Nr4a1 governs the influx and inflammatory output of the early subset, and its deletion worsens healing [37]. Recruitment is only half the story. The heart contains resident macrophages of embryonic and fetal-monocyte origin maintained by local proliferation [38], distinguishable from monocyte-derived cells by CCR2 expression in mouse and human myocardium [39,40]. Embryonic-derived macrophages support angiogenesis and repair while monocyte-derived macrophages promote inflammation, and CCR2 blockade in the adult improved function [41]; selective depletion of the self-renewing resident compartment worsened peri-infarct remodeling and could not be compensated by recruited cells [42]; and resident CCR2+ macrophages drive monocyte recruitment while resident CCR2− macrophages restrain it [39]. Reporting macrophage content without separating origin and state discards the variable that carries the biology. The inadequacy is not merely conceptual: macrophages conditioned by a therapeutic cell product were reported to adopt a phenotype that was neither M1 nor M2 [14].

### 4.4. Efferocytosis and the Transition to Resolution

The switch from clearance to resolution is the best-defined causal node in the sequence. Macrophage MerTK mediates uptake of apoptotic cardiomyocytes, and *Mertk* deficiency produced delayed resolution, larger infarcts and worse systolic function [43]. The pathway is regulated at the level of receptor integrity: proteolytic cleavage of the *MerTK* ectodomain generates a soluble decoy, and mice expressing a cleavage-resistant receptor had improved repair after ischemia–reperfusion injury [44]. A distinct macrophage state marked by high Trem2 expression emerges late in the infarct, and administration of soluble Trem2 improved structure and function, showing that the efferocytic program is manipulable from outside the cell [45]. Failure at this node produces secondary necrosis, persistent danger signaling and a poorly consolidated scar; success releases IL-10 and TGF-β and licenses angiogenesis and controlled fibrogenesis.

### 4.5. Adaptive Regulation, Fibrogenesis and Chronic Remodeling

Depletion of Foxp3+ regulatory T cells impaired healing while their expansion improved it, operating through monocyte and macrophage differentiation rather than direct cardiomyocyte protection [46]. The relevant clones are antigen-specific: myosin heavy chain alpha emerged as a dominant epitope and transferred MYHCA-specific CD4+ T cells acquired a regulatory phenotype and mediated cardioprotection [47]. Conventional dendritic cells are required for adequate healing, since conditional ablation caused infarct expansion, sustained inflammation and impaired neoangiogenesis [48], whereas B cells modify the response in the opposite direction through CCL7-dependent mobilization of Ly6C-high monocytes, so that B-cell depletion limited injury [49]. The sign of these effects is not fixed across disease stage: in established ischemic cardiomyopathy regulatory T cells convert to a dysfunctional, proinflammatory state and their depletion improves remodeling [50]. The terminal phase couples immunity to stroma, and human multiomic analyses of infarcted and failing myocardium have resolved zone-specific myeloid and fibroblast states and identified an immune–fibroblast signaling circuit whose interruption in mice reduced fibrosis [51,52].

### 4.6. Complement, Platelets and the Edges of the Current Map

Two arms of the early response are markedly less resolved than the myeloid sequence. Complement activation follows ischemic membrane injury immediately and was once the most attractive target in acute infarction; its clinical test was decisive in the negative direction, since C5 inhibition in a large trial of primary percutaneous intervention did not reduce mortality [53]. That trial is more informative than a bare null result, because target engagement was documented rather than assumed: in a pharmacodynamic subset of 229 patients, 90% of those receiving pexelizumab achieved more than 70% inhibition of hemolytic complement activity at baseline and at 24 h. Incomplete inhibition therefore cannot carry the explanation, which strengthens rather than weakens the negative result. Pathway redundancy and mistimed intervention remain open, and no cardiac study has since tested complement with the genetic precision applied to nucleic-acid sensing. Platelets are present from the first minutes and are the most heavily manipulated compartment in clinical practice, yet their contribution to the repair phase, as distinct from thrombosis and microvascular obstruction, has not been isolated by lineage-restricted perturbation. Both are treated here as incompletely mapped rather than as established checkpoints, and no part of the convergence argument rests on them.

### 4.7. The Problem of Translating Time

Murine phase boundaries are sharp and reproducible; human boundaries are inferred from circulating cell kinetics and cross-sectional tissue, since serial myocardial sampling is not possible. The human single-cell and spatial atlases are snapshots taken at surgery or autopsy [51,52]: they establish that the murine cell states have human counterparts without establishing when those states appear or how long they persist in an individual patient. Interspecies scaling of these intervals has not been measured. A therapy whose mechanism depends on acting inside a two-to-four-day window therefore faces an unquantified risk of being administered outside it, and no biomarker currently tells an investigator which phase a given patient occupies. What is established here is the murine sequence and its causal nodes; what remains inferred is its human timing.

## 5. Causal Immune Checkpoints Shaping the Repair Trajectory

Not all pathways described above carry equal weight. This section retains those for which necessity, sufficiency or rescue has been demonstrated, since only these can support a mechanistic claim about how a therapy works. For each we state the endogenous role, the strongest causal experiment, the structural consequence, the therapeutic relevance and the principal limitation.

### 5.1. Spatially Restricted Danger Sensing

Cytosolic DNA sensing converts cardiomyocyte death into a transcriptional program that recruits and activates myeloid cells. Necessity has been shown by germline and conditional deletion of cGAS, STING, IRF3 and IFNAR across two independent laboratories and both permanent-occlusion and reperfusion models [29,30], and the cellular source has been assigned to border-zone cardiomyocytes by cell-type-restricted *Irf3* deletion [31]. The structural consequence of an unrestrained pathway is reduced border-zone integrity and increased rupture risk. Therapeutic relevance is direct: augmenting the cytosolic-DNA exonuclease TREX1 in macrophages using a synthetic noncoding RNA reduced scar size after ischemia–reperfusion injury [54]. The limitation is that interferon signaling is also required for antiviral surveillance and no study has defined the therapeutic window in an aged or comorbid host.

### 5.2. Neutrophils as Effectors and Instructors

The endogenous role is dual and temporally separated. The strongest causal experiments remain antibody depletion, which worsened outcome and blocked reparative macrophage conversion [33], and independently, blockade of von Willebrand factor-dependent endocardial entry, which reduced monocyte influx and also worsened function [35]; the S100A8/A9–marrow axis provides a blockable amplification loop that does not require eliminating the cells [34]. The structural consequence of neutrophil loss is a fibrotic, poorly organized infarct. This checkpoint argues against depletion strategies and in favor of selective interruption of amplification. Antibody depletion is not lineage-clean, and the timing of depletion relative to reperfusion differs between studies.

### 5.3. Resident and Recruited Macrophage Trajectories

Embryonic-derived resident and monocyte-derived recruited macrophages perform partly opposing functions [38,41]. Necessity has been shown for the resident compartment by selective depletion [42] and for the recruited compartment by inhibition of monocyte recruitment [41], with the two resident subsets themselves exerting opposite effects on recruitment [39]. Two caveats belong in the same breath. The recruited-compartment result was obtained in a genetic cardiomyocyte-ablation model rather than coronary occlusion, and the angiogenic consequences attributed to the resident compartment were described in neonatal recovery; the depletion experiments in adult ischemic hearts measured remodeling and bulk fibrosis, not vascular architecture. Human corroboration exists at the level of subset identity and origin [40,51], not of subset function. The limitation that matters most for therapeutic inference is that CCR2 targeting is blunt: it removes a population that also performs necessary phagocytic work, and the benefit of blockade is strongly timing-dependent—as the von Willebrand factor experiment shows, reducing CCR2-positive influx at the wrong moment is harmful [35].

### 5.4. Efferocytosis and Immune-Metabolic Reprogramming

Uptake of apoptotic cells is not disposal but reprogramming of the engulfing macrophage. Necessity is established for MerTK by genetic deletion [43] and refined by the cleavage-resistant receptor experiment, which isolates receptor integrity rather than receptor expression as the operative variable [44]. Sufficiency of external manipulation is supported by soluble Trem2 administration [45]. Efficient efferocytosis accelerates the inflammatory-to-reparative transition and improves scar consolidation. It is a biologically plausible candidate convergence node and a priority for platform-specific causal testing, but the evidence that the therapeutic platforms act through it is uneven and in places absent. The necessity and sufficiency results above come from endogenous injury biology, not from any therapeutic agent. For selected cell products, benefit is lost after myeloid perturbation, although the depletion is not lineage-selective and efferocytosis itself was not the perturbed step. For vesicle preparations the link is inferential: enhanced phagocytic capacity has been measured in primed macrophages, but no cardiac study has blocked efferocytosis and shown loss of vesicle benefit. For biomaterials there is no intervention-level efferocytosis evidence at all. Calling it a common target would therefore overstate what has been shown. The unresolved limitation is quantitative: no study has established how much additional efferocytic capacity would be needed to change a human infarct.

### 5.5. Regulatory Lymphocytes and Network Control

Regulatory T cells appear to act as network controllers rather than as direct effectors, since their principal demonstrated action is on monocyte and macrophage differentiation [46]. Adoptive transfer establishes sufficiency. Findings obtained with IL-10-deficient regulatory T cells support a contribution of Treg-derived IL-10, but do not by themselves establish complete necessity or mediation: the IL-10-deficient arm failed to improve outcomes relative to saline rather than being compared directly against wild-type Tregs, and its donors differed from the other arms in sex and in sorting strategy [55]. Antigen specificity has been mapped to a cardiac myosin epitope [47]. Dendritic cells sit upstream [48] and B cells oppose [49]. The dominant limitation is stage dependence: the same cell type is protective early and injurious in chronic ischemic cardiomyopathy [50], so no therapy targeting this checkpoint can be developed without a defined window.

### 5.6. Immune–Fibroblast and Immune–Vascular Coupling

The final checkpoint is the transduction step converting an immune state into tissue architecture, and it is the one on which the convergence argument ultimately rests. Macrophage-derived signals alter fibroblast activation and matrix content, an effect demonstrated within the cell therapy paradigm itself, where the induced macrophage response was accompanied by changed fibroblast activity, reduced border-zone matrix and improved passive mechanics of the infarct [10]. The macrophage-to-fibroblast step was perturbed directly, but only ex vivo: CCR2+ and CX3CR1+ macrophages sorted from infarcted hearts at seven days and co-cultured with cardiac fibroblasts had opposite effects, the former raising smooth muscle α-actin, lysyl oxidase and collagen 1α2 and the latter lowering them while raising connective tissue growth factor [10]. That establishes that the two subtypes instruct fibroblasts differently; it does not establish that this step mediates the mechanical outcome in vivo. In failing human myocardium a definable immune–fibroblast communication axis was identified and its interruption in mice reduced fibrosis [52]. Fibroblast-directed cellular immunotherapy provides the cleanest proof that the immune system can be instructed to remodel cardiac stroma: transiently engineered, in vivo generated chimeric antigen receptor T cells targeting fibroblast activation protein reduced fibrosis and restored function [56]. That experiment produces no new cardiomyocytes and is a useful conceptual boundary marker—immune-directed repair in its purest form.

### 5.7. What Is Not Yet a Checkpoint

The checkpoints above were selected because a perturbation experiment exists. Several frequently invoked mechanisms do not meet that standard in cardiac injury. Immune metabolism is the clearest case: metabolic reprogramming after engulfment of a therapeutic product has been demonstrated in lung [57] and altered macrophage state after cytosolic DNA sensing has been described in myocardium [30], but no cardiac study has shown that blocking a specific metabolic transition abolishes the benefit of a regenerative intervention. Pro-resolving lipid mediator programs are equally plausible and equally untested in this therapeutic context. Chemokine axes other than CCL2/CCL7–CCR2 are described abundantly and perturbed rarely. Presenting such pathways at the same confidence as MerTK-dependent efferocytosis or CCR2-dependent recruitment is the most common evidentiary error in this literature, and it is why the convergence figure distinguishes arrow types rather than simply drawing connections.

## 6. Proximal Divergence: How Each Platform First Engages the Host

**Figure 2:**
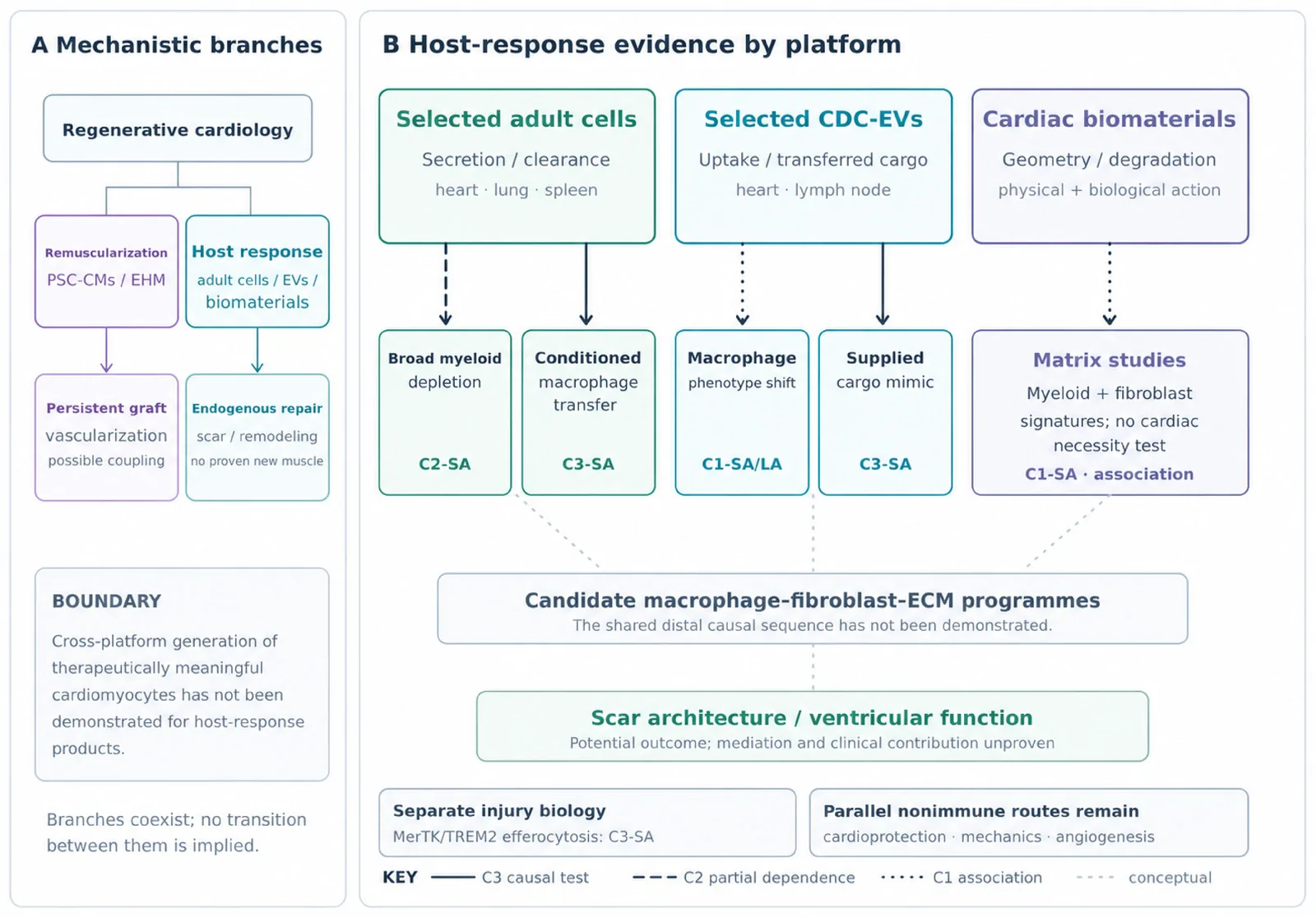
Platform-specific evidence map. (**A**) Remuscularization and host-response engineering coexist; cross-platform cardiomyocyte generation has not been demonstrated. (**B**) Solid, dashed and dark dotted arrows encode C3 intervention evidence, C2 partial pathway dependence and C1 association for the stated claim; gray dotted links are conceptual. Selected adult-cell experiments show loss of benefit after broad myeloid depletion and sufficiency of conditioned-macrophage transfer [14]. Selected cardiosphere-derived extracellular-vesicle (EV) experiments show associated macrophage phenotype changes and sufficiency of supplied candidate cargo [65,66], without proving endogenous cargo necessity or EV-mediated efferocytosis. Biomaterial studies map myeloid and fibroblast changes without testing cardiac immune necessity [75,77]. MerTK/TREM2 efferocytosis [43,44,45] belongs to endogenous injury biology. Distal convergence and clinical mediation remain hypotheses. SA, small animal.

The platforms are frequently discussed as though they were three formulations of one therapy. They are not. Their first interactions with the injured heart are mechanistically unrelated, and only downstream do they begin to look alike. This section describes the divergence; Section 7 describes what converges and what does not. The resulting three-tier causal map is shown in Figure 2.

### 6.1. Adult Cell Products: Disposal Before Secretion

The first proximal event for a cell product may not be secretion but disposal. This is a proposition rather than a finding, and it must be disaggregated, because at least six distinct proximal mechanisms have been proposed and they are not mutually exclusive: active secretion by viable cells; apoptosis or other regulated death of the administered cells; efferocytosis and phagocytic clearance of those cells; pulmonary or splenic sequestration with signaling from a distant compartment; local sterile immune activation caused by the injection itself; and genuine persistence. Different products, routes and models engage different combinations, and no single disposal pathway should be attributed to the class.

Two mechanistic chains are comparatively complete and are worth dissecting element by element. In the first, intravenously delivered human mesenchymal stromal cells embolize in the lung and the cardiac benefit in mice was attributed to TNF-α-stimulated gene 6 protein secreted by those pulmonary-trapped cells: knockdown of TSG-6 in the donor cells removed the improvement in fractional shortening and ejection fraction, establishing necessity of the mediator. Recombinant TSG-6 reduced infarct size but by significantly less than the cells themselves and was never tested against a functional endpoint, so it reproduces the effect only partially and sufficiency is not established [58]. Localization is unambiguous and unexpected—the active site is extracardiac. What the study does not establish is that TSG-6 acts through an immune-cell state change in the myocardium, or that this mediator accounts for the effects of other stromal-cell products. In the second chain, cardiosphere-derived cells given twenty minutes after reperfusion in rats lost their cardioprotective effect when macrophages were depleted, establishing immune dependence, and macrophages conditioned by those cells reproduced the benefit and reduced cardiomyocyte death on adoptive transfer, establishing sufficiency of the conditioned cell [14]. Localization is cardiac and the temporal ordering is correct. What it does not establish is which molecular signal conditions the macrophage, nor that the same dependence holds for other products or later treatment times.

Death of the administered cells as an active mechanism rests on evidence that is strong but extracardiac. Recipient cytotoxic cells render infused stromal cells apoptotic by a perforin-dependent route, and in perforin-deficient recipients both the apoptosis and the immunosuppression were lost; the requirement for recipient cytotoxic activity could be replaced by infusing stromal cells rendered apoptotic ex vivo, and recipient phagocytes that engulfed them produced indoleamine 2,3-dioxygenase. In sixteen patients with steroid-resistant graft-versus-host disease, the five who responded were those whose circulating cells showed high cytotoxic activity against the product [59]; deletion of the apoptotic effectors BAK and BAX prevented cell death and attenuated therapeutic activity, with efferocytosis of the dying cells altering metabolic and inflammatory programs in alveolar macrophages [57]; and monocytes that phagocytose the product acquire a regulatory phenotype and redistribute from the lung [60]. Reprogramming of host macrophages by stromal-cell-derived prostaglandin E2 toward IL-10 production was shown to be necessary in a sepsis model [61]. All four experiments are extracardiac. They establish a mechanism for the class of product, not for the heart, and the cardiac equivalent—showing that blocking efferocytosis of an administered cell product abolishes cardiac benefit—has not been performed. That is a specific, addressable gap rather than a general caveat.

The compartment question cuts across all of this. Intravenous stromal cells improved left ventricular function with negligible cardiac homing, an effect attributed to systemic anti-inflammatory action [62], which locates the mechanism outside the heart even though the readout is cardiac. Whether systemic and local routes engage the same downstream macrophage program is unresolved and is one of the more consequential open questions in the field.

### 6.2. Extracellular Vesicles: Biodistribution, Uptake and Cargo

The reparative responses attributed to mesenchymal stromal cell-derived vesicles after ischemia–reperfusion injury, and the cargo catalog supporting them, have been reviewed comprehensively elsewhere and are not recapitulated here [28]; what follows is restricted to the evidence bearing on cross-platform convergence. Vesicles offer the possibility of linking biological activity to discrete molecular cargoes, although the administered preparations themselves frequently remain heterogeneous and incompletely defined. The separation method determines what else is present—lipoproteins, protein aggregates, free nucleic acid—and reporting standards exist precisely because preparations described by the same name are not comparable across laboratories [63]. Much of the cardiac literature describes preparations as “exosomes” without establishing endosomal origin; current guidance requires the generic term unless biogenesis has been demonstrated. Several influential cardiac studies predate those standards [64,65,66] and their conclusions should be read as conclusions about a vesicle-enriched conditioned-medium fraction.

Biodistribution constrains interpretation further. Labeled vesicles accumulate predominantly in liver, spleen and lung, with route and producer cell determining the pattern [67], and whether enough material reaches the infarct to account for the observed effect is rarely measured. Dose is generally expressed as particle number or protein mass, neither of which corresponds to the quantity of the implicated cargo delivered to a macrophage in the infarct.

Against that background the causal claims must be separated link by link. For cardiosphere-derived vesicles, benefit, macrophage uptake and associated phenotype changes were demonstrated, and miR-181b-loaded vesicles together with PKCδ-suppressed macrophages were sufficient to reproduce the effect [65]. That study also moved one step toward necessity: vesicles transfected with an antagomir to miR-181b lost most of their protective effect, which lifts the claim above bare sufficiency. It stops short of cargo necessity in the strict sense, because the study did not establish that antagomir transfection preserved vesicle number, size distribution and non-cargo composition unchanged; the effect was blunted rather than abolished, and no experiment deleted miR-181b in the donor cell. The design that would settle it has been performed once in this program for a different cargo: vesicles were harvested from donor cells transfected with a miR-146a hairpin inhibitor, and the resulting miR-146a-depleted vesicles protected cardiomyocytes less well than control vesicles but still suppressed apoptosis significantly [64]. That result is in vitro only and was never carried to an infarct endpoint, yet its direction is instructive: depleting the single most enriched cargo attenuates rather than abolishes the effect, which is what a multi-component vesicle would predict. The same asymmetry applies to the Y RNA fragment EV-YF1, which raised macrophage IL-10 and reduced infarct mass when injected as a naked oligoribonucleotide, with IL-10 neutralization abolishing protection in coculture [66]. Here the authors state the boundary themselves: establishing EV-YF1 as a mediator of vesicle benefit would require selective depletion of the fragment from the preparation, which they could not achieve without disturbing the remaining contents. Two further observations from that study cut against a purely macrophage-mediated reading—the fragment was also modestly cytoprotective for cardiomyocytes with no macrophages present, and the macrophage and apoptosis endpoints rest on three animals per group. A separate stromal-cell preparation attenuated ischemia–reperfusion injury through miR-182-regulated macrophage polarization, and there the donor cells themselves were transfected with a miR-182 inhibitor so that the harvested vesicles carried less of the implicated cargo; the resulting shift in macrophage phenotype was partially negated rather than abolished [68]. That depletion was read out on cultured macrophages alone and never carried to an infarct endpoint, and vesicle yield, size distribution and non-cargo composition were not reported as unchanged, so it too stops short of endogenous cargo necessity in the heart. Blockade of vesicle secretion in the donor cells does establish that the vesicle fraction is necessary for the parent cell therapy [64], which is a different and weaker claim than necessity of any given cargo.

One study comes closer to a complete chain than any other cell-free work in the heart. Intrapericardially delivered stromal-cell vesicles drained to the mediastinal lymph node were taken up by MHC class II-positive antigen-presenting cells, activating a PP2A–Akt–Foxo3 axis that induced IL-10, IL-33 and IL-34, expanded regulatory T cells and improved repair, with *Foxo3* knockdown abolishing the effect [69]. Each link was measured and the compartment in which the immune event occurs was identified rather than assumed. It is worth noting that the operative compartment was a lymph node.

Large-animal work diverges instructively. In pigs, cardiosphere-derived vesicles reduced scar and improved function when delivered intramyocardially but were ineffective when intracoronary [70]. That comparison is more granular than a simple route dichotomy. Microvascular obstruction fell after both routes, whereas scar size, cardiomyocyte apoptosis and CD45+ leukocyte infiltration in the infarct and border zones fell only after intramyocardial delivery. The intracoronary route therefore did engage something measurable without converting it into structural benefit, which is the same engagement-without-outcome pattern seen in the human trials of Section 9.3. Alongside this, vesicles from human-induced pluripotent-stem-cell-derived cardiac cells improved recovery without increasing arrhythmia [71]. Neither tested immune necessity. Separately, and importantly for the bifurcation, vesicles from human iPSC-derived cardiovascular progenitors improved function in chronically infarcted mouse hearts while dual lineage-tracing and mosaic analysis systems showed no generation of new cardiomyocytes [72]. A companion study of the same class of vesicles characterized the accompanying immune response: intramyocardial delivery three weeks after infarction reduced cardiac pro-inflammatory Ly6C-high monocytes and circulating IL-1α, TNF-α and IFN-γ, and in acute infarction reduced pro-inflammatory macrophages, Ly6C-high monocytes and neutrophils while raising IL-10; the same preparation showed low immunogenicity, provoking neither allogeneic T-cell responses nor natural killer cell activation nor immunoglobulin induction on repeated injection into immunocompetent animals [73]. No immune population was depleted in either study, so the immune claims remain associative. Cargo, however, was manipulated in one of them: co-delivery of an anti-miR-133 antagomir partially abrogated the functional benefit [72]. That establishes partial cargo dependence and nothing more—the antagomir arm is a loss-of-function test only, the authors did not test a miR-133 mimic, and the abrogation was partial, so the cargo is contributory rather than sufficient. The companion immunological study contains no cargo manipulation at all [73].

### 6.3. Biomaterials: Degradation, Mechanics and Foreign-Body Response

The three host-response platforms are not mechanistically equivalent and evidence obtained for one does not transfer to another. Cell products may act through secretion, persistence or clearance of a living agent, with a contribution from cell death established only in particular settings; vesicle preparations deliver heterogeneous, preparation-dependent molecular cargo with no autonomous behavior; biomaterials act through bulk physical properties, degradation kinetics and geometry, and persist long after any cell product would have disappeared. Their evidentiary situations differ accordingly: causal perturbation exists for cell products, partial cargo perturbation for vesicles, and association only for biomaterials in the heart. Biomaterials are the only platform with an irreducibly physical mode of action, and the one where automatic immune attribution is most tempting and least justified. Seven distinct mechanisms must be kept apart: deliberate immune instruction; nonspecific foreign-body response; mechanical restraint and wall-stress reduction; retention and delivery of a co-administered agent; signaling by degradation products; direct fibroblast and matrix effects; and demonstrated immune mediation, which is not a synonym for any of the preceding six.

Degradation products of extracellular matrix drive macrophages toward a constructive phenotype in vitro and promote progenitor migration and myogenesis [74], placing the degradation step rather than the intact scaffold at the center of the biological effect. In the heart, decellularized myocardial matrix injected after infarction altered transcriptional programs consistent with reduced inflammation and improved metabolic and muscle gene expression while improving hemodynamics [75], and a catheter-deliverable formulation improved ejection fraction, ventricular volumes and regional wall motion three months after transendocardial delivery in pigs [76], on six treated animals against four heterogeneous controls, with one treated animal lost within 24 h of injection and no background heart-failure pharmacotherapy in either arm. Higher-resolution profiling has since resolved which cell types and infarct regions the signals originate from, and higher marker-gene expression in a tissue-resident macrophage subcluster has been read as preservation of that compartment, alongside fibroblast, vascular and lymphatic changes [77]. The inference rests on transcript signatures rather than on cell counts or fate mapping, and that study reported no functional endpoint at all. All of this is mechanistic mapping and association; none of it has been shown to mediate the functional effect. No published cardiac biomaterial study has, to our knowledge, combined the material with macrophage depletion or a lineage-restricted genetic manipulation to test immune necessity. A product-specific macrophage depletion or lineage-restricted perturbation, with appropriate material and geometry controls, would test an individual immune-necessity claim; it would not establish the complete proposed pathway.

Two constraints police the interpretation. Geometry alone determines the intensity of the foreign-body response across chemically unrelated materials in rodents and non-human primates [78], so any immune effect attributed to composition must be controlled for size, shape and stiffness; in that work macrophage depletion sharply reduced deposition on the responsive geometry, so the size effect is itself macrophage-driven rather than passive [78]. And the most rigorous demonstration that a scaffold instructs rather than merely irritates comes from skeletal muscle, where the pro-regenerative microenvironment required CD4 T cells: functional recovery was lost in *Rag1*-deficient hosts and restored by transfer of wild-type but not *Rictor*-deficient CD4 T cells [79]. The IL-4 receptor arm is weaker than it is usually reported to be, because *Il4ra*-deficient animals were assayed for macrophage CD206 expression and not for functional recovery, and on a different genetic background from the rest of the study. The material system also improved function in a small uncontrolled human cohort [80]. This cross-organ evidence cannot be transferred to myocardium without qualification and defines an experimental standard that cardiac biomaterial studies have not met.

## 7. Distal Convergence and Its Limits

### 7.1. The Macrophage–Fibroblast–Extracellular-Matrix Axis

If the platforms diverge proximally, the case for treating them together must rest on what they share downstream. The macrophage–fibroblast–extracellular-matrix axis is a candidate shared endpoint: individual links are experimentally tested within some preparations, while cross-platform mediation remains untested. For adult cell products, experiments have linked macrophage responses to altered fibroblast activity, reduced border-zone matrix content and improved passive infarct mechanics: functional benefit was lost after macrophage-related perturbations in one model [10] and partly in another [21], while broad myeloid depletion abolished benefit and conditioned-macrophage transfer reproduced it in a third [14]. For vesicles, macrophage uptake and associated phenotype changes are shown for cardiosphere-derived and cardiovascular-progenitor preparations [65,73]; broad systemic myeloid depletion reduced benefit from a separate mesenchymal-stromal-cell exosome preparation [68]. Supplying miR-181b reproduced benefit in the rat CDC-vesicle model [65], but no cardiac study has established necessity of endogenous cargo. For biomaterials, the entire platform is a matrix intervention, and the myeloid and fibroblast responses have been mapped with spatial resolution, though without perturbation [75,77]. Independently of therapy, the same axis carries causal weight: a definable immune–fibroblast circuit in failing human myocardium can be interrupted in mice with reduced fibrosis [52], and directing lymphocytes against activated fibroblasts is sufficient to remodel cardiac stroma and improve function [56].

The convergence is therefore real but bounded, and the boundaries should be stated whenever the axis is invoked. It is strongest at the level of recurring cell states and tissue-remodeling programs rather than at the level of defined molecular pathways. It is supported to different degrees across platforms: causally for selected links in selected cell products, partially for selected vesicle preparations, and associatively for biomaterials. Causal support attaches to individual links, not to the axis as a whole. Convergence at this level is not evidence that the proximal mechanisms are interchangeable, and it is not evidence that all therapeutic benefit is immune-mediated, since the nonimmune routes of Section 8 remain available for every platform. It is substantially less secure in humans than in experimental models, where it rests on cross-sectional tissue correspondence rather than perturbation [51,52]. The axis is best stated as one on which platforms can act, not as a mechanism they are known to share.

### 7.2. What Does Not Converge: Cardiomyocyte Proliferation

A claim of convergence on cardiomyocyte renewal would be the most commercially attractive and the least supported statement this review could make. No cross-platform convergence on therapeutically meaningful cardiomyocyte generation has been demonstrated. Therapeutically meaningful cardiomyocyte generation was not detected in the best-controlled adult-cell and extracellular-vesicle studies and remains untested or insufficiently tested for many biomaterial platforms. Lineage tracing after adult cell therapy detected no new cardiomyocytes despite functional benefit [10]. For one vesicle preparation the test was done prospectively: in chronically infarcted hearts, two independent reporter systems—MerCreMer/ZEG and mosaic analysis with double markers—showed that vesicles from human iPSC-derived cardiovascular progenitors did not generate new cardiomyocytes while still improving function [72]. For biomaterials no comparable lineage experiment exists. What has been reported is a discrete band of cardiac muscle along the endocardium—the surface at which the material was delivered—occupying a significantly larger fraction of the infarct in treated pigs than in controls, identified by trichrome staining with troponin-T confirmation on adjacent sections [76]. The authors themselves read it as possibly “a combination of preserved cardiomyocytes and newly formed cardiomyocytes”. Without lineage tracing that reading cannot be resolved, and salvage, migration and sampling at the delivery surface remain live alternatives. Cardiomyocyte generation is therefore not established for any biomaterial.

Four propositions must be kept apart here, and only the first three are defensible from the present evidence: absence of detected cardiomyocyte generation in a specific experiment; evidence against cardiomyogenesis for a particular preparation in a particular model; absence of demonstrated cross-platform convergence; and universal biological impossibility. The last does not follow, since failure to detect cardiomyogenesis with particular products, doses, timings and detection thresholds cannot establish that no adult-cell, vesicle or biomaterial preparation could ever achieve it. What can be said is that the convergence has not been demonstrated, and that this absence is what most cleanly separates host-response engineering from the remuscularization branch [7,8,9].

The neonatal heart supplies the instructive contrast. Macrophage depletion in neonatal mice abolished regeneration and neoangiogenesis after cardiac injury and produced a fibrotic scar instead [81], which shows that immune cells can be required for true regeneration when the myocardial substrate is permissive. The adult post-infarct substrate is far less permissive, and the same immune machinery therefore appears to favor repair rather than regeneration. The distinction is between what the immune system is capable of supporting and what the adult cardiomyocyte is capable of doing.

### 7.3. Route Dependence as a Translational Hypothesis

One observation recurs across platforms and deserves promotion from methodological footnote to explanatory hypothesis. Adult cell products rejuvenated the infarcted rodent heart after direct intramyocardial injection but not after vascular infusion of the identical product [82]. Cardiosphere-derived vesicles reduced scar and improved function in pigs when delivered intramyocardially and were ineffective by the intracoronary route [70]; in that study leukocyte infiltration and cardiomyocyte apoptosis tracked the effective route rather than the delivered dose, which is the closest existing link between route and a host-response readout. Intravenous stromal cells, by contrast, act only through a systemic route, with negligible cardiac homing [16,62], and the clearest intravenous mechanism identified to date operates from the lung [58].

Read together, these results suggest that intramyocardial delivery may not be a more efficient version of intracoronary or intravenous delivery but a mechanistically different intervention. Needle injection into injured myocardium creates a localized sterile perturbation—tissue disruption, local danger signal release, high local concentration of the agent—that intracoronary infusion does not reproduce even if the same number of cells or particles is administered. If that is correct, then clinical trials using intracoronary or intravenous delivery tested a biologically different intervention rather than a weaker version of the same one, which would reframe a substantial part of the negative clinical record.

Timing behaves in the same way and is not separable from route. Bone marrow mononuclear cells delivered by the same intramyocardial route into an established chronic scar produced no functional benefit, although they still induced local macrophage accumulation [24]; and within a single cardioprotection study, cardiosphere-derived cells infused twenty minutes after reperfusion reduced infarct size whereas the same cells infused after a two-hour delay did not [14]. The window, not merely the needle, is part of the intervention.

The hypothesis must be stated with its evidentiary status attached. The rodent comparison was a direct within-study contrast [82] but was reported as a research letter with a limited endpoint set; the porcine comparison [70] and the intravenous studies [58,62] are cross-study contrasts differing in species, product, dose and timing simultaneously. A randomized large-animal route comparison does exist and complicates a general preference for intramyocardial delivery. Pigs with reperfused infarction were randomized to catheter-based intramyocardial vesicles, intracoronary vesicles or vehicle; the intracoronary group differed from vehicle for infarct volume at one month while the intramyocardial group did not; a difference between those two significance tests is not itself a direct route-to-route comparison. A companion randomized placebo-controlled study reported lower infarct volume and higher ejection fraction after intracoronary delivery at three months [83]. Three qualifications keep this from settling the question in the opposite direction. The intracoronary arm received twice the particle dose of the intramyocardial arm, so route and dose are confounded. The early disadvantage of the intramyocardial arm was attributed by the authors to needle injury, a procedural harm rather than a biological property of the route. And the product differed from the cardiosphere-derived preparation used in the contrasting porcine study. These results motivate direct dose-matched route comparisons with procedural controls; they do not isolate a route effect or establish general superiority of either route. Route dependence remains a mechanistic hypothesis requiring experimental validation, not an established principle, and the studies that bear on it measured no immune endpoint at all.

### 7.4. Non-Independence of the Supporting Evidence

Apparent replication in this field is frequently not independent, and the aggregate impression of convergence is generated by fewer experimental systems than the citation count suggests. Three clusters matter specifically. The cardiosphere-derived cell and vesicle program—macrophage necessity and sufficiency [14], vesicle-borne miR-181b [65], EV-YF1 and IL-10 [66], vesicle secretion blockade [64], the porcine route comparison [70], the clinical CADUCEUS and ALLSTAR program [84,85] and the synthetic Y RNA that upregulates TREX1 [54]—originates from one laboratory and one product line, with overlapping author teams and overlapping preparations. Readers should note in particular that the danger-sensing therapeutic result of Section 5.1 and the vesicle results of Section 6.2 are not independent of one another. The cardiovascular-progenitor vesicle work comprises two companion publications from a single program addressing different questions in related models [72,73]. The result that reframed the field and the route-dependence experiment that qualified it come from the same group [10,82], and the most complete cell-free platform-to-outcome chain rests on a single study [69]. None of this impugns the individual results, several of which are technically exemplary, and repeated investigation within a program is a legitimate and often necessary activity. It is simply not replication by an independent group, and the two must not be counted alike. The next highest-value experiments are replications in unrelated hands rather than extensions in the same ones.

### 7.5. Dose, Preparation and Product Identity

A mechanistic claim about a biological product is only as stable as the product. Cell products vary with donor age, passage, tissue source and cryopreservation, which alter both secretory output and susceptibility to host-mediated killing—and susceptibility to killing is precisely the variable that the extracardiac mechanistic work identifies as decisive [57,59]. A product optimized for viability at release may therefore be optimized against its own proposed mechanism of action. Vesicle preparations vary with the separation method and carry non-vesicular co-isolates [63]. Failure to replicate a cardiac result across laboratories is therefore uninformative about the mechanism unless product equivalence has been demonstrated, and that condition has almost never been met.

## 8. Where Convergence Ends: Competing Mechanisms and Falsification

Several nonimmune mechanisms explain parts of the same data and are not weaker candidates because they are less fashionable.

**Direct cardiomyocyte cytoprotection:** Vesicle preparations reduce cardiomyocyte death in vitro without any immune cell present, and effects on infarct size measured within the first 72 h are as compatible with cytoprotection as with altered repair [66,68]. Infarct size and repair quality are different endpoints and are routinely conflated.

**Angiogenesis and vascular support:** Improved border-zone perfusion can preserve function independently of scar composition. Angiogenic effects are documented for all three platforms and are not, by themselves, immune effects; several vesicle and matrix preparations act directly on endothelium.

**Matrix mechanics and ventricular restraint:** The clearest example is alginate hydrogel, developed on an explicitly mechanical rationale and associated with improved peak oxygen consumption in a randomized trial with no immune endpoint whatsoever [86]. A material can change chamber mechanics without instructing any immune cell, and this comparator should be retained wherever a biomaterial immune claim is made.

**Retention and exposure:** Scaffolds increase residence time of co-delivered cells or vesicles, so an apparent material effect may be a pharmacokinetic effect on a different agent [76].

**Electrical stabilization:** Scar architecture and border-zone conduction influence arrhythmic burden and thereby function; the large-animal vesicle study that assessed arrhythmia found none but was not designed to test the electrical mechanism [71].

**Genuine replacement:** Pluripotent-derived cardiomyocytes and engineered heart muscle do generate contractile tissue, and the boundary of the present argument lies exactly there [7,8,9].

Immune and nonimmune routes are not mutually exclusive, and demonstrating macrophage dependence for one preparation does not establish macrophage dependence for a class. The convergence hypothesis is compatible with parallel operation. What it is not compatible with is the finding that immune perturbation is dispensable.

### Falsification

The hypothesis is worth holding only if it can fail. The following results would weaken or refute it, and each is experimentally attainable with existing methods.

F1: Preserved efficacy despite abolition of the proposed pathway. Retained benefit after clodronate or genetic depletion of monocytes and macrophages, or in CCR2-deficient recipients, with depletion verified in the infarct at the relevant time. The converse has been shown for two products [10,14], so a well-powered negative replication in independent hands would be directly contradictory.

F2: Loss of efficacy explained by something other than pathway necessity. A depletion experiment that abolishes benefit but also alters biodistribution, retention or systemic toxicity has not demonstrated necessity of the pathway. Depletion arms must therefore report agent retention and off-target morbidity, which most do not.

F3: Structural benefit without measurable immune modification. Improved infarct mechanics or scar compaction in the absence of any detectable change in myeloid composition, state or function, using assays of adequate sensitivity.

F4: Immune target engagement without a corresponding measured structural or functional gain. In VCU-ART3, interleukin-1 blockade reduced systemic inflammatory markers after ST-elevation infarction without changing echocardiographic ventricular volumes or ejection fraction; infarct size was not measured as an outcome [87]. Engagement is therefore separable from the ventricular outcomes measured in that trial.

F5: Failure across independently produced preparations. If the mechanism holds only for one product line and cannot be reproduced with an independently manufactured preparation of the same nominal class, the claim is about a product, not a mechanism [63].

F6: Potency correlating with a nonimmune property. If lot-to-lot potency in vivo tracks a mechanical, angiogenic or cytoprotective assay but not the proposed immune assay, the immune pathway is a correlate rather than the mediator.

F7: Apparent state transitions that are actually population changes. A shift in marker proportions can arise from recruitment, selective survival or dilution rather than from reprogramming of individual cells. Fate mapping, clonal barcoding, proliferation and death measurements and trajectory analysis distinguish these possibilities; flow-cytometric proportions alone do not [11,38,42].

Two further falsifiers are platform-specific and complete the list.

F8: Vesicles: retention of benefit when myeloid uptake is selectively blocked while cardiomyocyte uptake is preserved; or loss of benefit when the implicated cargo is depleted from donor cells with vesicle yield, size distribution and non-cargo composition verified unchanged. The second experiment has not been reported for any cardiac vesicle preparation.

F9: Biomaterials: equivalent improvement in infarct mechanics and ventricular function in immunodeficient hosts, or with an immunologically inert material of matched geometry, stiffness and degradation rate. Because geometry alone governs the foreign-body response [78], a matched-geometry inert control is the minimum acceptable comparator and is almost never included.

## 9. Human Translation: Efficacy Without Mechanistic Closure

Human evidence must be interrogated with three separate questions, because a trial can answer the first while remaining silent on the others. Did treatment improve a prespecified endpoint? Did treatment change the intended immune target, at the intended time, in the intended compartment? Did that change mediate the structural or clinical effect?

### 9.1. Efficacy

The strongest single statement about efficacy in the host-response branch comes from individual-patient-data meta-analysis rather than from any one trial. Pooling 1252 patients from twelve randomized trials of cell therapy after acute myocardial infarction, the ACCRUE collaboration found no benefit for clinical events, ejection fraction or ventricular volumes [88]. That is E3-H for the absence of demonstrated efficacy across the included trials and C0-H for any mechanistic claim, since the constituent trials measured no immune endpoint; a negative efficacy result does not identify the mechanism of failure. Individual trials are consistent with it. CADUCEUS, a phase 1 study of autologous cardiosphere-derived cells, reported reduced scar mass and increased viable myocardium without improvement in ejection fraction [84], while the larger randomized ALLSTAR trial of allogeneic cardiosphere-derived cells did not reduce scar size at six months [85]. ALLSTAR should be read as inconclusive rather than definitively negative. The sponsor stopped it at a prespecified interim analysis when the conditional probability of rejecting the primary null had fallen to 1.1%, so the twelve-month magnetic resonance primary endpoint was never completed, and the authors state that the truncated trial was not powered for the primary or secondary endpoints at six months either. Its secondary results are also not uniformly null: end-diastolic volume, end-systolic volume and natriuretic peptide were each lower with treatment at six months (*p* = 0.02 for all three), which the authors read as disease-modifying bioactivity and which the same power argument obliges us to treat as exploratory. BAMI closed with recruitment and event rates far below assumption and cannot support a group comparison [89]. CHART-1 did not meet its primary endpoint [90]. CONCERT-HF, a phase 2 trial, reported a reduction in a composite heart-failure event endpoint with cardiac cells (6.5% versus 28.1%, *p* = 0.043); heart-failure hospitalization considered alone did not reach significance (3.2% versus 21.9%, *p* = 0.053). Quality of life improved with stromal cells, and neither ejection fraction nor scar changed. No endpoint was adjusted for multiplicity [91]. POSEIDON established safety and reported changes in chamber geometry rather than ejection fraction [92]. The SCIENCE trial of allogeneic adipose-derived stromal cells in chronic ischemic heart failure was safe but showed no improvement in end-systolic volume, ejection fraction, symptoms, walk distance, natriuretic peptide or C-reactive protein [93]. DREAM-HF, the largest randomized trial in this space, did not meet its primary endpoint of time to recurrent decompensated heart-failure events or resuscitated symptomatic ventricular arrhythmias (hazard ratio 1.17, 95% confidence interval 0.81–1.69), and its hierarchical secondary endpoints were neutral as well. Two prespecified findings run the other way and should not be suppressed: in the 537-patient analysis population the composite of myocardial infarction or stroke was reduced (cause-specific hazard ratio 0.42, 95% confidence interval 0.23–0.76), and ejection fraction rose modestly relative to control at twelve months (*p* = 0.041), an effect confined to patients with elevated baseline C-reactive protein. The three-point major adverse cardiovascular event composite was a post hoc exploratory analysis and did not reach conventional significance [13]. One clinical result now sits outside this pattern, and it belongs to the other branch. The engineered-heart-muscle cohort described in Section 3.2 reported increases in target wall thickness, ejection fraction and symptom score at three months [17]. It is uncontrolled and interim, so it establishes neither efficacy against a comparator nor mechanism; it documents wall thickening during treatment with a remuscularization product; without a control group, the treatment-attributable magnitude and comparisons with adult host-response products remain unresolved. Two observations after immunosuppression withdrawal may inform questions about graft persistence. In one patient, withdrawal of immunosuppression was followed by donor-specific antibodies above 20,000 mean fluorescence intensity, a strongly positive complement-dependent cytotoxicity test and a fall in ejection fraction from 39% to 28%; in another, withdrawal produced a weak antibody response, a negative cytotoxicity test and preserved function. These uncontrolled observations do not constitute a graft-ablation experiment and cannot establish whether graft persistence mediates functional change.

Biomaterial translation is earlier and more heterogeneous. VentriGel, a decellularized myocardial matrix hydrogel, was delivered transendocardially to fifteen post-infarction patients in an uncontrolled first-in-man study reporting safety, feasibility and improvements in functional class and walk distance [94]. Alginate hydrogel is the instructive outlier: it improved peak oxygen consumption in a randomized comparison on an explicitly mechanical rationale, with no immune endpoint measured or intended [86]. A field arguing that biomaterials act through immune instruction has to account for the fact that its only randomized functional success was designed on the opposite premise.

### 9.2. Circulating Target Engagement, and What It Is Not

Directly localized myocardial immune engagement after a regenerative intervention has not been shown in patients. Circulating biomarker changes have been reported and require narrower interpretation. A prespecified secondary analysis of CONCERT-HF measured plasma biomarkers at baseline and at days 7, 30 and 180 in 96 of the 125 randomized patients, samples being unavailable for the remaining 29, and reported selective, time-dependent modulation of inflammatory and matrix-remodeling proteins including TNF-α, pentraxin-3, PDGF-BB, GDF-15, BMP-9 and MMP-1 after stromal cells, cardiac cells or their combination [95]. The denominator differs from that of the parent trial and should be reported as 96 of 125 rather than as the trial population [91].

The defensible formulation is therefore that circulating target engagement has been reported, without evidence that these changes represent cardiac immune reprogramming or causally mediate clinical benefit. Six things must be kept apart, and this study establishes only the first. Circulating biomarker modification is a change in plasma protein concentration. Cardiac localization would require showing that the change originates in or reflects the myocardium. Cellular target engagement within myocardium would require demonstrating an altered immune-cell state in cardiac tissue. Structural modification would require an imaging or histological endpoint. Causal mediation would require the correct temporal sequence with a prespecified mediation model and competing mediators estimated jointly. Clinical benefit is a separate endpoint again, and the parent trial was a phase 2 study not powered for it. No published study in this field has linked more than two of these six.

A pattern runs through this section: a mismatch between the mechanism proposed for a therapy and the endpoint chosen to evaluate it. Trials built on a paracrine or immunomodulatory rationale have been judged on ejection fraction and time-to-event composites measured months to years after a transient intervention, while the biology invoked to justify them operates over days and in a compartment none of these trials sampled [13,88,93]. Aligning the readout with the hypothesis is a design problem rather than a biological one, and it is addressed in Section 10.

Baseline inflammatory status occupies a different logical position and is frequently misdescribed. In DREAM-HF the treatment effect was reported as concentrated among patients with elevated baseline high-sensitivity C-reactive protein [13]. The subgroup deserves more credit than post hoc findings usually receive, because the analysis and the 2 mg/L threshold were both prespecified, the threshold having been carried across from CANTOS. What it still cannot do is establish the mechanism. A baseline covariate identifies who responded, not what the therapy changed; C-reactive protein was never remeasured on treatment as an engagement readout, and no mediation analysis was performed. It is a candidate endotype marker whose correct use is prospective stratification.

The three-level distinction of Section 2.2 is what the human data most obviously lack. Circulating immune changes after treatment have been documented; changes within the myocardial immune compartment have not been measured in any of these trials; and causal mediation of a cardiac outcome by either has not been tested. The available human evidence is therefore insufficient to establish that immune modulation mediates the observed therapeutic effects, and the review does not claim that it does.

### 9.3. Anti-Inflammatory Trials as External Mechanistic Controls

Pharmacological anti-inflammatory trials are not regenerative therapies and are not equivalent to the three platforms. They are retained here as external contextual comparators and nothing more. They establish that inflammation is a modifiable contributor to cardiovascular outcomes in humans; they do not demonstrate that cell, extracellular-vesicle or biomaterial therapies act through the same pathway, and no inference from these trials to the mechanism of a regenerative platform is licensed. Their value is as human experiments measuring different parts of a possible immune-to-outcome sequence. No trial cited here measures and establishes the full mediation chain from cardiac immune engagement through structural modification to clinical benefit. Their lesson is double-edged. Canakinumab in CANTOS reduced recurrent cardiovascular events in patients with prior infarction and elevated C-reactive protein, establishing that modulating a single cytokine axis changes human cardiovascular outcomes [96], without any claim of regeneration or repair. The result is narrower than it is usually rendered. Only the 150 mg dose met the prespecified multiplicity-adjusted threshold (hazard ratio 0.85, 95% confidence interval 0.74–0.98); the 300 mg dose did not, despite a nominally similar estimate. All-cause mortality was unchanged (hazard ratio 0.94, 95% confidence interval 0.83–1.06), and fatal infection was more frequent with canakinumab than with placebo. Suppressing an innate pathway therefore carries a measurable host-defense cost, which is the clinical counterpart of the interferon dilemma set out in Section 5.1. Tocilizumab given at primary percutaneous intervention in ASSAIL-MI increased the myocardial salvage index on cardiac magnetic resonance and reduced microvascular obstruction [97]. Its evidentiary status must be stated as four separate claims. The structural treatment effect is E3-H, limited to the randomized magnetic resonance endpoint actually tested. Systemic immune target engagement is C1-H, supported by direct measurement of a circulating biomarker after treatment. The term is reserved in this review for cases of that kind, in which the relevant immune pathway, cell population, mediator or tissue response was actually measured after the intervention; where the evidence is associative, histological or extrapolated from related biology, the text says “associated immune change”, “findings consistent with immune modulation”, “putative engagement” or “candidate mechanistic link” instead. Causal mediation of the structural benefit by that engagement is C0-H, because no mediation analysis was performed and randomization to treatment cannot demonstrate that a biomarker change lay on the causal path. Cardiac cellular localization and myocardial immune reprogramming are also C0-H, because neither was measured. The qualification that matters most lies downstream: the C-reactive protein area under the curve was substantially lower with tocilizumab (*p* < 0.001), so engagement is not in doubt, yet final infarct size (7.2% versus 9.1%, *p* = 0.08), baseline-adjusted left ventricular volume (*p* = 0.54) and six-month natriuretic peptide (*p* = 0.25) did not differ. It is therefore the closest existing example of an immune intervention with a measured immune target and a measured myocardial structural readout in the correct temporal order, and it has not been shown to mediate that structural change. Anakinra reduced the systemic inflammatory response after ST-elevation infarction in VCU-ART3, with a C-reactive protein area under the curve of 67 versus 214 mg·day/L (*p* < 0.001) [87]. It is often cited as dissociating engagement from infarct size, but that trial did not measure infarct size as an endpoint: imaging was echocardiographic, and the enzymatic estimate appears only as a baseline characteristic. What it does show is engagement without a change in ventricular volumes or ejection fraction. Against these sit unambiguous failures: C5 inhibition did not reduce mortality [53], and high-dose colchicine at reperfusion did not reduce infarct size on cardiac magnetic resonance [98].

Two conclusions follow. Reducing an inflammatory biomarker is not automatically reparative and does not by itself demonstrate immune-guided tissue repair. And the trials that failed were, with few exceptions, suppression trials rather than redirection trials—consistent with the experimental record showing that untimed suppression impairs scar formation [26,27] and that blocking early recruitment worsens function [33,35].

### 9.4. Why Translation May Fail for Reasons Unrelated to the Thesis

Several structural mismatches operate independently of whether the mechanism is correct. Rodent studies are performed almost exclusively in young, male, metabolically normal animals, whereas human infarction occurs in older patients with diabetes, obesity, chronic kidney disease and clonal hematopoiesis, each altering myeloid biology at baseline; the sex restriction is explicit even in recent high-quality work [55]. Reperfusion status determines the shape of the inflammatory curve, and contemporary door-to-balloon times compress the window in which an early immune intervention could act. Route and timing differ systematically between preclinical and clinical studies, and the route-dependence hypothesis of Section 7.3 implies that intracoronary and intravenous trials may have tested a different intervention. Disease stage compounds this: for bone marrow mononuclear cells the preclinical benefit was obtained during early infarct maturation and was absent in chronically scarred hearts [24], whereas CHART-1, CONCERT-HF, DREAM-HF, SCIENCE and POSEIDON all enrolled patients with established ischemic cardiomyopathy [13,90,91,92,93]. Transient mechanisms are evaluated against endpoints measured months to years later, with no intermediate measurement capable of showing whether the intended biology ever occurred.

### 9.5. Comparative Translational Maturity

The three platforms are at different stages, and treating them as a single class distorts the picture in both directions. Adult cell therapy has the largest clinical dataset and the weakest human mechanistic case, with the first circulating engagement data only now appearing [95]. Biomaterials have the smallest clinical dataset and the clearest demonstration that benefit can be real without being immune [86,94]. Therapeutic vesicles have the strongest preclinical causal evidence [65,66,69] and no completed randomized cardiac efficacy trial, which places them in the unusual position of being the best-supported mechanism and the least tested therapy. The platform with the cleanest mechanistic hypothesis is thus the one still early enough to be designed around the mechanism from the outset.

## 10. Designing and Falsifying Immune-Guided Repair

If these therapies are developed as host-response engineering rather than as replacement, most elements of the development plan change. What follows is a specification, not an aspiration.

**Product identity and potency:** Potency assays should measure the intended host activity—induction of a defined macrophage state, enhancement of efferocytic capacity, fibroblast-modulating activity, or susceptibility of the product to phagocytic clearance—rather than viability, marker panels or particle count. For cell products this implies assaying apoptosis susceptibility, since apoptosis is required for immunomodulation in noncardiac models [57,59]. For vesicles it implies MISEV2023-compliant characterization with explicit reporting of non-vesicular co-isolates [63]. For biomaterials it implies reporting geometry, stiffness and degradation rate alongside composition [78].

**Dose and biodistribution:** Exposure at the target compartment must be measured rather than assumed, given hepatic and splenic sink effects for vesicles [67] and pulmonary entrapment for intravenous cells [16,58].

**Route and timing as design variables:** Route should follow the route that worked preclinically, and timing should be specified relative to reperfusion and to the efferocytosis window rather than by logistical convenience [70,82].

**Patient endotyping:** Enrichment by inflammatory phenotype is defensible as a selection strategy and should be prespecified and multiplicity-controlled rather than discovered post hoc, which is the correct use of the DREAM-HF observation [13].

**Serial immune phenotyping with a proximal compartment:** Peripheral monocyte subset and activation profiling before treatment and at defined early time points, paired where feasible with a compartment closer to the heart such as coronary sinus blood or pericardial fluid, since plasma alone cannot localize the effect [95].

**Functional rather than descriptive immune assays:** Ex vivo phagocytosis and efferocytosis assays on patient monocytes, because transcriptional state descriptions do not establish function and the efferocytic checkpoint carries the strongest causal support [43,44,45].

**Imaging endpoints matched to the mechanism:** Myocardial salvage index and microvascular obstruction early [97]; infarct heterogeneity, extracellular volume, border-zone extent and scar compaction rather than ejection fraction late, since the preclinical effect is on infarct mechanics and matrix content [10].

**Prespecified longitudinal mediation with competing mediators:** Treatment, mediator and tissue outcome measured in the correct temporal order, with immune and nonimmune routes estimated jointly rather than sequentially, so that a mechanical or angiogenic mediator can compete on equal terms [86].

**Preclinical validation in relevant hosts:** Aged, female and comorbid animals, and large-animal models with clinically realistic reperfusion times, before further phase 3 commitment.

**Independent replication and built-in falsification:** At least one arm per program designed to remove the proposed mechanism and show loss of benefit, and at least one replication in a laboratory with no stake in the product [10,14].

What this means in practice for trial design can be stated compactly. Endpoints should match the claimed mechanism rather than default to ejection fraction: a remuscularization claim requires graft survival and mass, electromechanical integration and regional contractility, whereas a host-response claim requires scar architecture, border-zone composition and matrix organization. Immune profiling should be spatial and serial rather than systemic and single-timepoint, since the compartment in which a change occurs is the point at issue. Pathway-specific perturbation, not global immunosuppression, is what converts an association into a mechanism. Durability and safety require follow-up beyond the window in which a transient agent could still be acting. And circulating biomarkers are worth measuring only where a myocardial correlate has been established, because a systemic signal that has never been localized cannot support a cardiac mechanistic claim however reproducible it is.

## 11. Conclusions

Regenerative cardiology is usefully read as two mechanistic families, and conflating them has cost the field a decade of interpretive clarity. The division is an organizing device proposed here rather than an established classification, and the evidence supporting it is heterogeneous and platform-dependent. True remuscularization is feasible in primates: pluripotent-derived cardiomyocytes and engineered heart muscle form persisting, vascularized grafts with evidence of coupling [7,8,9]. Human evidence now includes an uncontrolled cohort [17] and graft histology from one explanted heart [9]; clinical efficacy and contractile contribution remain unproven. Meaningful remuscularization has not been demonstrated for adult stromal-cell products, extracellular vesicles or biomaterials, whose observed effects require other explanations.

The strongest defensible version of that explanation is the following. For selected interventions in preclinical models, observed benefit may reflect a temporally and spatially bounded redirection of endogenous repair rather than myocardial replacement; the platforms diverge in their proximal engagement with the host and may affect, to different degrees, macrophage–fibroblast–extracellular-matrix programs. For adult cell products this rests on loss of benefit after myeloid depletion, reproduction of benefit by non-cellular sterile stimuli, and reproduction of benefit by transfer of conditioned macrophages [10,14,21]. For selected vesicle preparations, the evidence spans macrophage uptake or phenotype shifts [65,73], broad-depletion sensitivity in an MSC-exosome model [68], T-regulatory-cell perturbation in an intrapericardial model [69], and sufficiency of supplied candidate cargo [65,66]. These experiments involve different products and models; endogenous cargo necessity has not been shown for any of them. For biomaterials the cardiac evidence remains associative and the causal evidence is cross-organ [77,79]. Convergence on this axis is therefore causally established for individual links in individual preparations, not across the branch, and it is markedly less secure in humans than in experimental models.

The overclaim to be avoided is that immune reprogramming is the universal or exclusive mechanism of regenerative cardiac therapy, or that it has been demonstrated in humans. Neither is true. Mechanical restraint, direct cytoprotection, angiogenesis and retention explain parts of the same dataset, and the only randomized human trial in this space with a positive functional endpoint was built on a purely mechanical rationale [86]. No cross-platform convergence on therapeutically meaningful cardiomyocyte generation has been demonstrated: it was not detected in the best-controlled adult-cell and vesicle studies [10,72] and remains insufficiently tested for biomaterials, which is a statement about the present evidence rather than about biological impossibility.

Three unresolved links must be acknowledged wherever this framework is used. The first is functional rather than descriptive characterization of the therapeutically induced macrophage state, which has been described transcriptionally in many studies and measured functionally in almost none. The second is the absence of a cardiac experiment showing that clearance of the administered agent is required for cardiac benefit, the evidence for that step being entirely extracardiac [57,59,60]. The third is the absence of prespecified human mediation analysis: circulating engagement is now documented [95], cardiac localization is not, and mediation has not been attempted.

Stated plainly: a causal host-response mechanism underlying clinical regenerative benefit has not been demonstrated in humans. Immune modulation by these platforms is well documented in animals and partially documented in patients, but documentation of modulation is not demonstration of mediation. The shared distal pathways set out in Section 7 are best read as partially supported nodes and, in the case of efferocytosis and the biomaterial arm, as hypotheses. What would change this is specifiable rather than aspirational: cardiac necessity experiments for agent clearance, cargo-depletion rather than cargo-mimic experiments for vesicles, a matched inert-geometry control for biomaterials, and at least one clinical trial designed from the outset around serial immune measurement in a proximal sampling compartment with prespecified mediation analysis. The framework organizes that agenda; it does not itself validate any mechanism (Box 1).

Box 1 **Evidence standard for decisive extracellular-vesicle studies.**

**Applied to every vesicle study cited in **Section 6 and Section 7**. A study that does not satisfy items 6–10 supports an EV-associated effect rather than a vesicle–cargo mechanism, and is described as such in the text.**

1. Source cell identity, culture conditions and depletion of serum vesicles.

2. Separation and concentration method, with orthogonal confirmation.

3. Particle and protein dose, route, timing, and measured biodistribution rather than assumed delivery [67].

4. Positive and negative vesicle markers, and assessment of common co-isolates including lipoproteins and protein aggregates [63].

5. Whether endosomal origin was established; if not, “extracellular vesicle” or “small-EV-enriched preparation” rather than “exosome”.

6. EV-depleted conditioned-medium and non-vesicular fraction controls.

7. Cargo abundance per particle or per active dose, not expression enrichment in the donor cell.

8. Donor-cell cargo knockout or loading-pathway perturbation, with vesicle yield, size distribution and non-cargo composition verified unchanged.

9. Recipient-cell uptake and target engagement demonstrated in the tissue of interest, not only in culture.

10. Necessity, sufficiency and rescue tested as separate experiments; replication in a second laboratory, model, sex or species.

**None of the cardiac vesicle studies cited here satisfies item 8. Cargo sufficiency has been shown repeatedly; endogenous cargo necessity has not been shown for any cardiac preparation.**

## Data Availability

No new data were created or analyzed in this study. Data sharing is not applicable to this article.

## Supplementary Materials

The following supporting information can be downloaded at: https://www.mdpi.com/article/10.3390/biology15201884/s1, Table S1: Decisive mechanistic studies, classified claim by claim; Table S2: Human evidence, classified claim by claim.

## Abbreviations

APC, antigen-presenting cell; CAR, chimeric antigen receptor; CDC, cardiosphere-derived cell; CMR, cardiac magnetic resonance; DAMP, damage-associated molecular pattern; EHM, engineered heart muscle; EV, extracellular vesicle; FAP, fibroblast activation protein; G-CSF, granulocyte colony-stimulating factor; hsCRP, high-sensitivity C-reactive protein; IFN, interferon; IL, interleukin; I/R, ischemia–reperfusion; MI, myocardial infarction; MSC, mesenchymal stromal cell; PTX3, pentraxin 3; scRNA-seq, single-cell RNA sequencing; TGF-beta, transforming growth factor beta; Treg, regulatory T cell; vWF, von Willebrand factor.
