# Supplementary material for: Host-Response Engineering in Regenerative Cardiology: Mechanistic Boundaries Between Remuscularization and Immune Reprogramming
Source: Biology (Basel). 2026 Oct 19;15(20):1884. doi: 10.3390/biology15201884 (PMC13701815; doi:10.3390/biology15201884)
Supplement: Supplementary file 1 [file biology-15-01884-s001.zip › biology-4552497-supplementary.pdf]

Supplementary Materials

Host-Response Engineering in Regenerative Cardiology: Mechanistic Boundaries Between Remuscularization and Immune Reprogramming

Luca Bonanni and Nicola Ferri

Reference numbers in both tables correspond to the reference list of the main manuscript. The evidence codes and context tags are defined in Section 2.2 of the main manuscript; the abbreviations used in Tables S1 and S2 are listed in the abbreviation footnote below the tables. All sources cited in Tables S1 and S2 are also cited in the main manuscript and are included in its reference list. Reference numbers were renumbered in revision; they correspond to the reference list of the revised main manuscript.

Table S1. Decisive mechanistic studies, classified claim by claim.

Each row classifies one stated claim. The E axis (therapeutic efficacy), the C axis (proposed mechanism), the non-applicable designations E– and C–, and the context tags H, LA, SA and IV are defined in Section 2.2 and are not restated here. Two rules govern reading of the table: a C3 label applies only to the link actually tested, and label ranges are not used, so a study supporting several claims occupies several rows. The final column identifies studies that must not be counted as independent replication of one another.

**Study and identifier**	**Species/model (context)**	**Intervention, dose, route, timing**	**Claim classified**	**E category**	**C category**	**Causal perturbation**	**Outcome measured**	**Principal limitation**	**Independence warning**
Murry 2004 [2]; Balsam 2004 [3]. PMID:15034593; PMID:15034594	Mouse, infarct (SA)	Genetically tracked or purified hematopoietic stem cells; intramyocardial; acute	Adult hematopoietic cells do not transdifferentiate into cardiomyocytes (refutational observation)	E–	C–	Cardiomyocyte-restricted and ubiquitous reporter transgenes across 145 transplants, with an assay able to detect a single grafted cardiomyocyte; three separately purified donor populations in the companion study, plus a parabiosis arm testing circulating cells	No donor-derived cardiomyocytes and no increase in cardiomyocyte number versus sham. Donor cells expressed CD45 and Gr-1 and disappeared: 33.1 ± 19.9% of injected cells were detectable at 10 days and 0.2 ± 0.2% at 30 days	Refutes replacement only; silent on paracrine or immune benefit. C– because donor-cell fate, not a mechanism, was tested	Two independent laboratories — genuine concordance
van Berlo 2014 [4]. PMID:24805242	Mouse, development/ageing/injury (SA)	Endogenous Kit-lineage tracing; no therapeutic agent	Endogenous c-kit+ cells contribute cardiomyocytes at ~0.03% or less (quantitative lineage-tracing observation)	E–	C–	Inducible dual lineage tracing—a direct quantitative measurement of lineage output	Negligible myocyte contribution; larger endothelial contribution	Cre efficiency debated; single lineage marker. C– because the claim is quantitative rather than mechanistic	Independent of the c-kit therapeutic literature

Study and identifier	Species/model (context)	Intervention, dose, route, timing	Claim classified	E category	C category	Causal perturbation	Outcome measured	Principal limitation	Independence warning
Chong 2014 [7]; Liu 2018 [8]. PMID:24776797; PMID:29969440	Macaque, infarct (LA)	hESC-derived cardiomyocytes (~0.75–1 × 10^9); intramyocardial	Implanted cardiomyocytes persist, are vascularized and form host–graft adherens and gap junctions; graft–host electromechanical synchrony was demonstrated in [7] only (direct histological and imaging observation)	E–	C–	Graft-specific histology and imaging; GCaMP3 calcium imaging in [7]; histology alone in [8]; no perturbation of the graft in either	Large vascularized grafts. Ventricular arrhythmias occurred in all four cell-treated macaques in [7] and in one of six in [8], where arrhythmia burden did not differ statistically from vehicle	C– because graft persistence and coupling were observed, not tested as a mechanism. Ref. [7] reported no statistically significant effect on ejection fraction, attributing this to small group sizes and small infarcts	Two publications from one program (Murry laboratory)
Liu 2018 [8] (second claim). PMID:29969440	Macaque, infarct (LA)	As above	Remuscularization mediates the observed improvement in left ventricular ejection fraction	E2-LA (function improved versus controls)	C0-LA	None—no graft ablation, no dose–response for graft mass	Ejection fraction improved at 1 and 3 months	The contractile contribution of the graft was not isolated, so mediation by remuscularization is proposed rather than tested	Same program as [7]
Jebran 2025 [9]. PMID:39880949	Rhesus macaque (LA)	Epicardial EHM allografts, 40 × 10^6 cells per patch (1×) to 200 × 10^6 (5×, maximum feasible dose); tacrolimus + methylprednisolone	Claim 1—allografts are retained, persist histologically and are vascularized to 6 months (direct histological and imaging observation)	E– (not an efficacy claim)	C–	None—observational histology and magnetic resonance imaging	20 implanted macaques, 66 patches from 2.64 × 10^9 cells; retention to 6 months; no arrhythmia or tumour formation	Retention is not integration and vascularization is not electromechanical coupling; neither was demonstrated. C– because the observation does not test a mechanism	Single program
Jebran 2025 [9] (claim 2). PMID:39880949	Rhesus macaque (LA)	As above	Claim 2—the allograft produces a structural and functional treatment effect (dose-dependent wall thickening and improved regional and global function)	E2-LA, limited to the endpoints directly demonstrate d	C–	Randomization of chronic heart-failure macaques to control ($n = 7$), 2× EHM ($n = 3$) or 5× EHM ($n = 4$); one 5× EHM animal died in the post-anesthesia recovery phase from low cardiac output syndrome, leaving six evaluable treated animals, three per dose	Wall thickness +1.5 ± 0.8 mm (2×) and +2.2 ± 0.1 mm (5×) vs −0.03 ± 0.07 mm controls ($p = 0.0128$; $p < 0.00001$); thickening fraction +21 ± 0.2% vs +0.6 ± 3.3% ($p = 0.0039$); LVEF +7 ± 3% vs −2 ± 2% ($p = 0.0389$)	Very small cohort. Seven macaques were assigned to engineered heart muscle and six were evaluable; sustained contractility improvement occurred in three of those six and ejection-fraction improvement in three animals. Hosts were immunosuppressed	Single program

Study and identifier	Species/model (context)	Intervention, dose, route, timing	Claim classified	E category	C category	Causal perturbation	Outcome measured	Principal limitation	Independence warning
Jebran 2025 [9] (claim 3). PMID:39880949	Rhesus macaque (LA)	As above	Claim 3—graft persistence mediates the functional benefit	E–	C0-LA	None—no graft ablation and no graft-mass dose–response for function	Not tested	The proposed mechanism linking persistence to function was not experimentally isolated	Single program
Jebran 2025 [9] (human observation). PMID:39880949	One participant in the BioVAT-HF-DZHK20 Phase 1/2 first-in-human trial, bridge-to-transplant (H)	Epicardial EHM allograft from 400 × 10^6 cardiomyocytes and stromal cells (10× dose); heart transplantation 3 months after implantation	Grafted cardiomyocytes were retained and the graft was partially vascularized in the explanted human heart (direct histological observation)	E– (not an efficacy claim)	C–	None—histology of the explanted heart	Cardiomyocyte retention confirmed; graft capillary density 187 ± 5 vs 963 ± 12 per mm² in recipient myocardium; grafted cells smaller and less mature; connexin 43 sparse	One patient, not a compassionate-use case but a trial participant. Electromechanical integration, quantitative contractile contribution, clinical efficacy, generalizable safety and reproducibility were not established. No other participant contributed explant histology to this report; cohort outcomes were reported separately [17].	Single program
Vagnozzi 2020 [10]. PMID:31775156	Mouse, ischemia–reperfusion (SA)	Adult progenitors, freeze/thaw-killed cells or zymosan A; intramyocardial; at reperfusion or 1 week	A sterile innate stimulus is sufficient to reproduce the functional benefit of cell therapy	E2-SA	C3-SA (sufficiency of the stimulus)	Substitution of live cells by dead cells and by an acellular stimulus; cyclosporine A immunosuppression; clodronate macrophage depletion; Ccr2-null and Cx3cr1-null hosts	Reduced border-zone matrix, altered fibroblast activity, improved passive mechanics and ejection fraction; benefit persisted ≥8 weeks; saline injection caused mild inflammation without benefit	Male and female mice, but not randomized (the authors state that randomization was omitted because animals were genetically identical littermates); assessment blinded; local injection; one delayed timing	Same group as [82]
Vagnozzi 2020 [10] (second claim)	Mouse, ischemia–reperfusion (SA)	As above	No new cardiomyocytes were detected despite functional benefit (lineage-tracing observation)	E–	C–	Lineage tracing—a direct test of cardiomyocyte origin	No new cardiomyocytes detected	A detection-threshold statement about this product and model, not a general claim about cardiomyogenesis. C– because absence of detection is not a test of necessity or sufficiency	As above

Study and identifier	Species/model (context)	Intervention, dose, route, timing	Claim classified	E category	C category	Causal perturbation	Outcome measured	Principal limitation	Independence warning
Vagnozzi 2020 [82]. PMID:32202933	Rodent, infarct (SA)	Bone marrow mononuclear cells: 1×10^7 intravenously versus 1×10^6 intramyocardially (one-tenth the dose), 1 week post-I/R	The benefit is route-dependent and is not explained by dose	E2-SA	C2-SA	Direct within-study route comparison with tracked mTomato+ cells	Infusion gave no functional benefit: <0.2% of blood cells at 5 min and 2 days, <3 cells per mg tissue, ~450 cells in the whole heart, all within CD31+ capillaries. Intramyocardial delivery at one-tenth the dose improved systolic function and attenuated hypertrophy	Research letter; limited endpoints; single species. Argues against a systemic paracrine mechanism for this product	Same group as [10]
Vagnozzi 2021 [24]. PMID:34310160	Mouse, chronic infarct scar, male and female C57BL/6J (SA)	150,000 bone marrow mononuclear cells or saline; intramyocardial, two border-zone sites; 8 weeks after 120 min ischemia–reperfusion; echo 2 weeks later	Claim 1—bone marrow mononuclear cells delivered into an established chronic scar do not improve function	E2-SA (randomized, no benefit)	C–	Randomization to cells or saline at 8 weeks; concurrent 1-week cohort for comparison	No difference in fractional shortening, end-systolic volume, heart weight/body weight or border-zone fibrosis; cartilage oligomeric matrix protein (COMP; also known as thrombospondin-5) deposition raised in both 8-week groups (n = 4, 6, 9, 11 for COMP)	Only bone marrow mononuclear cells were tested—the authors state that other modalities remain to be examined. The 1-week comparator is a concurrent but previously published, reanalysed cohort, not a randomized stage allocation	Same group as [10,82]
Vagnozzi 2021 [24] (claim 2). PMID:34310160	As above (SA)	As above	The absence of benefit despite preserved local inflammation supports stage- or substrate-dependent treatment response	E–	C1-SA	Timing comparison with associative histological characterization; the scar substrate itself was not perturbed	Cells still induced local inflammation in the chronic scar (greater activated CD68+ macrophage content than saline at 3 days) yet produced no functional benefit; COMP-rich mature scar in late-stage infarcts	The comparison supports stage or substrate dependence but cannot assign causality to the mature scar microenvironment; baseline dysfunction, ceiling effects and the reanalysed comparator remain alternative explanations	Same group as [10,82]

Study and identifier	Species/model (context)	Intervention, dose, route, timing	Claim classified	E category	C category	Causal perturbation	Outcome measured	Principal limitation	Independence warning
de Couto 2015 [14]. PMID:26214527	Rat, ischemia–reperfusion (SA)	Cardiosphere-derived cells, 5 × 10^5 per 100 µl; intracoronary infusion 20 min after reperfusion (45 min ischemia); n = 4–6 rats per group	Broad myeloid depletion blocks cardioprotection by this CDC preparation	E2-SA	C2-SA (broad myeloid depletion)	Systemic clodronate depletion; benefit was lost after depletion	Reduced infarct size at 48 h with 20 min but not 120 min delivery; clodronate abolished protection	Clodronate depletion is broad and does not isolate a macrophage lineage; acute cardioprotection window; downstream steps remain untested	Same program as [65,66,64,70,84,85,54]
de Couto 2015 [14]. PMID:26214527	Rat, ischemia–reperfusion (SA)	Cardiosphere-derived cells, 5 × 10^5 per 100 µl; intracoronary infusion 20 min after reperfusion (45 min ischemia); n = 4–6 rats per group	Transfer of CDC-conditioned macrophages is sufficient to reproduce cardioprotection	E2-SA	C3-SA (transfer sufficiency only)	Adoptive transfer of CDC-conditioned macrophages; 20 min versus 120 min timing comparison	Conditioned macrophages reproduced protection and attenuated cardiomyocyte apoptosis	The conditioning signal was not identified; transfer sufficiency does not establish necessity of a molecular pathway or complete mediation	Same program as [65,66,64,70,84,85,54]
Ben-Mordechai 2013 [21]. PMID:23973704	Mouse, infarct (SA)	Bone marrow MSCs, mononuclear cells or saline into the infarct; clodronate liposome depletion 4 h before or 3 days after MI	Macrophages are partially required for the benefit of stromal-cell therapy	E2-SA	C2-SA	Timed macrophage depletion (4 h before or 3 days after infarction) with and without cell therapy; the macrophage compartment then recovered spontaneously, since depletion was transient and not repeated	Depletion attenuated but did not abolish the favourable effect on remodelling and function. Thirty-day mortality was 73% after depletion without cells and 50% with cells, versus 35% and 15% with macrophages intact. Only early depletion impaired function; late depletion did not	The authors conclude that only "some of the protective effects" are macrophage-mediated. Clodronate is not lineage-selective, no add-back rescue was performed, and the high mortality means the surviving-animal functional comparisons are subject to survivor bias	Independent group

Study and identifier	Species/model (context)	Intervention, dose, route, timing	Claim classified	E category	C category	Causal perturbation	Outcome measured	Principal limitation	Independence warning
Vasudevan 2023 [22]. PMID:37563727	Mouse, permanent ligation; C57BL/6J and Rag2-deficient hosts; male and female (SA)	Syngeneic neonatal GFP+ cardiomyocytes in Matrigel; four peri-infarct injections 3 days after ligation	A permissive CCR2 monocyte response is required for the pump-function benefit of a graft-based therapy, but not for the contractility benefit	E2-SA	C3-SA for the pump-function claim	Oral CCR2 inhibition (RS504393, 2 mg/kg twice daily, days 3–7) in wild-type mice, alongside the Rag2-deficient host comparison and single-cell profiling	Ejection fraction and wall-thickness benefit were lost under CCR2 inhibition, whereas contractility improvement persisted. Graft was detected in 3 of 7 wild-type and 1 of 7 Rag2-deficient hearts	Functional benefit was decoupled from graft survival—the authors note improvement "despite the relatively poor engraftment"—so this supports the host-response reading rather than remuscularization. Fibrosis and capillary density improved in neither strain. Pharmacological inhibition is not lineage-restricted, and the permanent-ligation model does not, in the authors' words, reflect "the most representative clinical scenario"	Independent group

Study and identifier	Species/model (context)	Intervention, dose, route, timing	Claim classified	E category	C category	Causal perturbation	Outcome measured	Principal limitation	Independence warning
Lavine 2014 [41]. PMID:25349429	Mouse; genetic cardiomyocyte ablation (Mlc2v-Cre × Rosa26-DTR, diphtheria toxin); neonatal and adult—no ischemic model (SA)	Liposomal clodronate 15 µL of 18 mg/mL intraperitoneally on postnatal days 1 and 5 (neonatal); CCR2 inhibitor RS 504393, 2 mg/kg twice daily by oral gavage (neonatal and adult)	Macrophages are required for neonatal cardiac repair, and blocking monocyte recruitment in the adult preserves the resident compartment and limits inflammation	E–	C2-SA	Clodronate depletion in neonates; pharmacological CCR2 inhibition in both age groups. No lineage-restricted genetic depletion, no reconstitution and no add-back arm	Neonatal depletion reduced cardiomyocyte proliferation, vascular density and endothelial proliferation and increased mortality, chamber dilation, hypertrophy and interstitial fibrosis. Adult CCR2 inhibition reduced inflammatory cytokines and neutrophil influx and preserved coronary microvascular density	The injury is genetic cardiomyocyte ablation, not infarction, so the result does not transfer to ischemic injury without qualification. Clodronate is not lineage-selective, and pharmacological CCR2 inhibition is not lineage-restricted, so broader depletion remains a live alternative—hence C2 rather than C3. No cardiac function, fibrosis or cardiomyocyte proliferation endpoint was reported for the adult CCR2-inhibition experiment. The authors note the "inherent limitations of studying macrophages in vitro" and that a contribution from macrophages in other tissues cannot be excluded	Independent group; related program to [42]

Study and identifier	Species/model (context)	Intervention, dose, route, timing	Claim classified	E category	C category	Causal perturbation	Outcome measured	Principal limitation	Independence warning
Dick 2019 [42]. PMID:30538339	Mouse; permanent coronary ligation (SA)	Endogenous compartments; lineage-restricted depletion, no exogenous agent	Resident and recruited macrophages are functionally non-interchangeable	E–	C3-SA for necessity only	Lineage-restricted depletion of TIMD4/Lyve1-defined resident macrophages, with fate mapping by parabiosis. No reconstitution or add-back arm	Divergent late remodelling: the effect of depletion appeared only between days 7 and 28, with no difference in infarct size or ejection fraction at day 7 and increased late mortality	Necessity is demonstrated, sufficiency is not: no resident population was reconstituted and the phenotype recovered. Angiogenesis and matrix architecture were not measured after depletion; only bulk fibrosis area was. Resident macrophages are 2–5% of infarct-zone macrophages, so the effect size is disproportionate to the compartment and the mechanism is not established	Independent group; related program to [41]
Bajpai 2019 [39]. PMID:30582448	Mouse; closed-chest ischemia–reperfusion, 90 min ischemia (SA)	Endogenous compartments; diphtheria-toxin-receptor depletion, no exogenous agent	Resident CCR2− and CCR2+ macrophage subsets exert opposite effects on monocyte recruitment and remodelling	E–	C3-SA for necessity only	CCR2-DTR and complementary genetic depletion of each resident subset. CCR2 was not pharmacologically blocked in this study and there was no add-back arm	Opposite effects of the two resident subsets on monocyte recruitment and on remodelling, with initial infarct size identical between genotypes	The authors state that the depletion strategies "are imprecise" and that "macrophage populations outside of the heart may be responsible for some of the observed phenotypes", and that the mechanistic basis "is likely multifactorial". Human subset function is inferred from expression phenotype [40]	Independent group
Horckmans 2017 [33]. PMID:28158426	Mouse, infarct (SA)	Endogenous neutrophils; depletion; NGAL immunodepletion and add-back	Neutrophil-derived NGAL contributes to reparative macrophage polarization and infarct healing	E–	C3-SA	Neutrophil depletion; NGAL immunodepletion from neutrophil secretome; add-back of secretome or recombinant NGAL	Depletion worsened function and fibrosis; secretome or recombinant NGAL restored a reparative macrophage phenotype	Antibody depletion is not lineage-clean; the study uses M1/M2/M2c categories	Independent group

Study and identifier	Species/model (context)	Intervention, dose, route, timing	Claim classified	E category	C category	Causal perturbation	Outcome measured	Principal limitation	Independence warning
Wünnemann 2025 [35]. PMID:41044418	Mouse, infarct (SA)	Endogenous leukocyte entry; functional von Willebrand factor blockade	Early vWF-dependent leukocyte entry through the endocardium supports infarct repair	E–	C2-SA	Functional vWF blockade with spatial multiomics	Impaired healing, more pronounced infarct thinning and deteriorated function on echocardiography at two weeks	von Willebrand factor has non-immune hemostatic and vascular roles, so the leukocyte-entry mechanism is not isolated; the authors note that blockade "might also affect primary hemostasis." Blockade only—no genetic deletion, no endocardium-restricted manipulation and no rescue	Independent group; distinct mechanism from [33]
Wan 2013 [43]; DeBerge 2017 [44]	Mouse; permanent ligation in [43], ischemia–reperfusion in [44]; human serum in [44] (SA/H)	Endogenous efferocytosis	MerTK-dependent efferocytosis is required for resolution and receptor integrity is the operative variable	E–	C3-SA	Germline and myeloid-restricted Mertk deletion; bone-marrow chimeras in both directions; a cleavage-resistant MerTK knock-in; CCR2 antagonism combined with Mertk deletion	Loss of efferocytosis, apoptotic cardiomyocyte accumulation, larger infarct and depressed systolic function; wild-type marrow transferred into Mertk-null mice restored systolic performance, and the cleavage-resistant knock-in reduced infarct size. Blocking monocyte recruitment reduced infarct size in wild-type but not in Mertk-null mice	Area at risk and day-3 infarct size were identical between genotypes, so the effect is not differential initial injury. Apoptotic cells eventually cleared even without MerTK, so other receptors contribute; a MerTK-agonist antibody alone did not induce IL-10 or suppress TNF-α. Soluble MerTK was elevated in human STEMI serum, which is association only	Two independent groups
Jung 2022 [45]. PMID:35933399	Mouse, infarct (SA); human tissue comparison	Soluble Trem2, 12 μg in a gelatin hydrogel injected into the peri-infarct myocardium at the time of ligation	The efferocytic program can be modified from outside the cell	E2-SA	C2-SA (sufficiency of an external ligand)	Administration of soluble Trem2 only. The single-cell and spatial profiling was performed on untreated infarcted mice, not on the treated cohort	Improved structure and function	Gain of function only: no Trem2 knockout, knockdown or depletion arm exists, so necessity is untested. The authors state that the cells affected by soluble Trem2 and the signalling involved remain to be determined	Independent group

Study and identifier	Species/model (context)	Intervention, dose, route, timing	Claim classified	E category	C category	Causal perturbation	Outcome measured	Principal limitation	Independence warning
King 2017 [29]; Cao 2018 [30]; Ninh 2024 [31]	Mouse, infarct (SA)	Endogenous cGAS–STING–IRF3–type I interferon	The pathway is required for adverse remodelling, rupture and death	E–	C3-SA	Germline deletion of Irf3, Ifnar, cGas and Sting; IFNAR-neutralizing antibody given 8–12 h and 48 h after ligation	Survival after permanent ligation rose from 61% in wild-type to 98% in Irf3-null and 100% in Ifnar-null mice, with less dilation and better contractile function; in [30] one-week mortality from free-wall rupture fell from 42% to 3%	Interferon signalling is also required for host defence and the therapeutic window is undefined. Sting-null mice showed only a non-significant mortality reduction, so other sensors probably contribute. In [30] the deletion is germline, and the authors state that whether the phenotype derives from myeloid cGAS "is unknown"	Three independent groups
Ninh 2024 [31]. PMID:39198639	Mouse, infarct; human infarct tissue (SA/H)	Endogenous IRF3; cell-type-restricted deletion	Border-zone cardiomyocytes, not infiltrating myeloid cells, are the dominant initiators of the interferon response	E–	C3-SA	Irf3-floxed mice crossed to cardiomyocyte-, fibroblast-, macrophage-, neutrophil- and endothelial-restricted Cre drivers; spatial transcriptomics and MERFISH	Only cardiomyocyte-selective Irf3 deletion abolished the clustered distribution of interferon-stimulated genes; of cells expressing Ifnb1 at day 3, 80% were border-zone cardiomyocytes and 20% border-zone fibroblasts	Cardiomyocyte deletion did not completely eliminate clustered expression, so other initiators contribute. CCR2-deficient mice, plasmacytoid-dendritic-cell-depleted mice and Irf3-null bone-marrow chimeras all retained the interferon foci, showing that bone-marrow-derived myeloid cells are not essential. No survival or remodelling endpoint was reported for the cell-restricted deletions	Independent group; distinct from [29,30]

Study and identifier	Species/model (context)	Intervention, dose, route, timing	Claim classified	E category	C category	Causal perturbation	Outcome measured	Principal limitation	Independence warning
TREX1 augmentation [54]	Mouse, ischemia–reperfusion (SA)	Synthetic noncoding RNA upregulating macrophage TREX1	Enhancing cytosolic DNA degradation in macrophages reduces scar size	E2-SA	C3-SA	TREX1 knockdown with siRNA; Trex1 overexpression; clodronate macrophage depletion; adoptive transfer of treated or Trex1-overexpressing macrophages; GW4869 blockade of vesicle release	Reduced scar size in rats and pigs. Knockdown of Trex1 blocked the effect, and macrophages were reported as both necessary and sufficient for it	Necessity was tested by transient knockdown rather than by germline or conditional deletion, and no cGAS or STING perturbation was performed, so the proposed upstream sensor is not itself tested. The authors state that the source of the damaged DNA and the mechanism linking clearance to reduced stress signalling remain undefined	Same program as the cardiosphere-derived cell and vesicle work [14,65,66]
Weirather 2014 [46]	Mouse, permanent coronary ligation and infarct models (SA)	Treg depletion and therapeutic T-cell activation	Regulatory T cells are required for infarct healing, and their therapeutic activation improves healing and survival	E2-SA	C3-SA	Foxp3-DTR ablation and anti-CD25 depletion; therapeutic CD28-superagonist activation 2 days after infarction	Ablation aggravated inflammation and raised mortality (day-7 survival 25% versus 55.9%); activation raised day-56 survival from 47.1% to 76.6% and reduced ventricular rupture from 23% to 5%, with increased de novo collagen in the scar	Depletion worsened outcome without altering collagen turnover, scar formation or rupture frequency, so the depletion and activation arms act through different proximal steps. Echocardiography in surviving animals showed no significant difference at day 7 or day 56, so the benefit is on survival and rupture rather than on function in survivors. Chronic ischemic cardiomyopathy reverses the direction of the Treg effect [50]	Independent group

Study and identifier	Species/model (context)	Intervention, dose, route, timing	Claim classified	E category	C category	Causal perturbation	Outcome measured	Principal limitation	Independence warning
Weirather 2014 [46] (second claim)	Mouse, permanent coronary ligation and infarct models (SA)	Treg depletion and therapeutic T-cell activation	The benefit is mediated by monocyte/macrophage differentiation	E–	C1-SA	None—macrophage phenotype was characterized but never perturbed, depleted or transferred	M1-like polarization after Treg ablation and M2-associated transcripts after activation; absolute monocyte numbers and Ly6C subset composition were unchanged	Mediation by the macrophage compartment is proposed, not tested. The unchanged monocyte numbers make a recruitment mechanism unlikely but do not establish the differentiation mechanism	Independent group
Rieckmann 2019 [47]. PMID:31408441	Mouse, BALB/c; permanent coronary ligation (SA)	Adoptive transfer of myosin-heavy-chain-α-specific CD4+ T cells (TCR-M), compared with ovalbumin-specific CD4+ T cells, one day before infarction	Antigen-specific CD4+ T-cell transfer preserves fractional area change; associated macrophage and collagen changes do not establish macrophage mediation	E2-SA	C1-SA	Adoptive transfer with an antigen-specificity control. The macrophage compartment was profiled but never perturbed	Fractional area change was better preserved at day 7 ($p < 0.05$). Infarct size, end-diastolic area, heart-to-body weight ratio and total cardiac leukocyte numbers did not differ, and end-systolic area did not reach significance ($p = 0.074$). Profibrotic MHC-II-high CCR2-low macrophages and infarct collagen content were both increased at day 5	Cells were transferred one day before infarction, so the design is prophylactic rather than therapeutic. No depletion, blockade or macrophage transfer was performed, so macrophage mediation is untested: the authors state that the mechanism "remains elusive" and only that it "might be dependent on" crosstalk with those macrophages. The same clone induces autoimmune myocarditis in donor mice	Independent group

Study and identifier	Species/model (context)	Intervention, dose, route, timing	Claim classified	E category	C category	Causal perturbation	Outcome measured	Principal limitation	Independence warning
Alshoubaki 2024 [55]. PMID:39090108	Mouse, infarct (SA)	Adoptive transfer of antigen-specific or IL-10-deficient Tregs	Transferred Tregs improve repair; the contribution of Treg-derived IL-10 is supported but not isolated	E2-SA	C2-SA	Adoptive transfer with an IL-10-deficient Treg comparison	Reduced fibrosis and cardiomyocyte death and improved function after transfer. IL-10-deficient Tregs did not significantly improve outcomes relative to saline	The IL-10 arm is a failure to reach significance against saline rather than a direct comparison against wild-type Tregs, so the loss of effect is not statistically established. Recipients were male but the IL-10-deficient donors were female and were sorted on CD4/CD25 rather than by Foxp3 reporter, an uncontrolled difference between arms. Among candidate mediators only nidogen-1 and IL-10 reduced Ly6C; annexin-A1 and galectins did not	Independent group
Lee 2009 [58]. PMID:19570514	Mouse, infarct; male NOD/SCID recipients (SA)	Human mesenchymal stromal cells; intravenous; acute	TSG-6 from pulmonary-trapped cells is required for the cardiac benefit and partially reproduces it	E2-SA	C2-SA	Transient siRNA knockdown of donor-cell TSG-6; recombinant TSG-6 given intravenously	Knockdown abolished the improvement in fractional shortening and ejection fraction. Recombinant TSG-6 reduced infarct size, but by significantly less than the cells themselves, and was never tested against a functional endpoint	Sufficiency is partial and untested for function, so "required and sufficient" overstates it. The recipients are immunodeficient, which constrains any adaptive-immune reading. The lung site is inferred from biodistribution, not perturbed: about 0.15% of cells reached the heart at 24 h and the authors state these "may also have contributed." Knockdown was transient and the scrambled control itself partially reduced TSG-6	Independent group

Study and identifier	Species/model (context)	Intervention, dose, route, timing	Claim classified	E category	C category	Causal perturbation	Outcome measured	Principal limitation	Independence warning
Galleu 2017 [59]. PMID:29141887	Mouse graft-versus-host disease (GvHD); human ex vivo assay (SA/IV, cross-organ)	Mesenchymal stromal cells; intravenous	Recipient cytotoxicity-induced apoptosis of administered stromal cells is required for immunosuppression outside the heart	E2-SA (non-cardiac disease model)	C3-SA/IV, cross-organ	Perforin-deficient recipients; infusion of ex vivo apoptotic stromal cells as rescue; recipient phagocyte depletion; pharmacological IDO inhibition	Loss and restoration of immunosuppression in a non-cardiac disease model. In 16 patients with steroid-resistant graft-versus-host disease the 5 responders were those whose cells killed the product most efficiently, assessed blind to clinical details	The corresponding cardiac necessity experiment has not been performed and cardiac relevance is C0. The human arm is a correlation in 16 patients with 17 doses, not an intervention	Independent group
Pang 2021 [57]. PMID:34764248	Mouse lung inflammation (SA/IV, cross-organ)	Mesenchymal stromal cells; intravenous; BAK/BAX manipulation	Intrinsic apoptosis of administered stromal cells is required for therapeutic immunomodulation outside the heart	E2-SA (non-cardiac disease model)	C3-SA/IV, cross-organ	Donor-cell BAK/BAX deletion	Loss of therapeutic activity when apoptosis was prevented	Lung model; cardiac relevance is C0	Independent group
de Witte 2018 [60]. PMID:29341339	Mouse, intravenous infusion, no cardiac injury (SA)	Mesenchymal stromal cells; intravenous	Infused stromal cells are phagocytosed by monocytes, which redistribute from lung to blood and liver bearing a regulatory phenotype	E–	C1-SA	Dual labelling to distinguish viable from dead cells, with cross-organ tracking; no perturbation	Phagocytosing Ly6C-low monocytes were present in lung, blood and liver	Tracking and phenotypic association only; no cardiac injury, no cardiac outcome and no mediation test	Independent group
de Witte 2018 [60] (second claim). PMID:29341339	Human monocytes, ex vivo (IV)	Human umbilical-cord stromal cells co-cultured with CD14+ monocytes	The monocyte phenotypic shift depends on uptake of the cells rather than on their secreted factors	E–	C2-IV	Conditioned medium compared with contact coculture, plus a 4 °C control that abolished uptake; primed monocytes then added to autologous mixed lymphocyte reactions	CD163 and CD206 rose only when uptake occurred (31.6 ± 3% versus 5.3 ± 2% with conditioned medium); primed monocytes raised Foxp3+ regulatory T cells from 8.9 ± 2% to 13 ± 2%	The 4 °C control is not selective for phagocytosis and conditioned medium also removes contact-dependent signalling, so uptake is implicated but not isolated; entirely extracardiac	Independent group

Study and identifier	Species/model (context)	Intervention, dose, route, timing	Claim classified	E category	C category	Causal perturbation	Outcome measured	Principal limitation	Independence warning
de Couto 2017 [65]. PMID:28411247	Rat and pig, ischemia–reperfusion (SA/LA)	CDC-derived exosomes after reperfusion; left-ventricular-cavity delivery with aortic cross-clamp in rats and direct intramyocardial injection in pigs	The vesicle preparation reduces infarct injury and is accompanied by macrophage phenotype changes in rat and pig models	E2-SA/LA	C1-SA/LA (association only)	CDC-derived exosomes compared with fibroblast exosomes or vehicle; macrophages profiled but not depleted in this report	Reduced infarct size in rat and pig models; altered macrophage abundance and polarization	Macrophage changes accompany benefit but do not establish necessity for this preparation; no macrophage loss-of-function arm was reported; pre-MISEV2023 nomenclature	Same program as [14,66,64,70]
de Couto 2017 [65]. PMID:28411247	Rat, ischemia–reperfusion (SA)	Fibroblast exosomes loaded with miR-181b or adoptive transfer of PKCδ-suppressed macrophages after reperfusion	Supplied miR-181b and PKCδ-suppressed macrophages are sufficient to reproduce protection	E2-SA	C3-SA	Cargo loading and adoptive transfer of PKCδ-suppressed macrophages	Reduced infarct size after supplied miR-181b or transfer of PKCδ-suppressed macrophages	Sufficiency of supplied cargo does not establish necessity of endogenous vesicular cargo	Same program as [14,66,64,70]
de Couto 2017 [65]. PMID:28411247	Rat, ischemia–reperfusion (SA)	CDC-derived exosomes transfected with miR-181b antagomir or scramble control; left-ventricular-cavity delivery under aortic cross-clamp after reperfusion	Antagomir perturbation attenuates protection, consistent with partial miR-181b-related pathway dependence	E2-SA	C2-SA	Antagomir to miR-181b transfected into the vesicle preparation before delivery; protection was blunted	Attenuated reduction in infarct size at 48 h	Pathway dependence, not cargo necessity: vesicle integrity and non-cargo composition after antagomir transfection were not documented, the effect was attenuated rather than abolished, and no donor-cell deletion was performed	Same program as [14,66,64,70]

Study and identifier	Species/model (context)	Intervention, dose, route, timing	Claim classified	E category	C category	Causal perturbation	Outcome measured	Principal limitation	Independence warning
Zhao 2019 [68]. PMID:30753344	Mouse, myocardial ischemia–reperfusion (SA)	MSC-derived exosomes injected into myocardium immediately after reperfusion; systemic clodronate liposomes before and after injury	Benefit from this MSC-exosome preparation is reduced after broad systemic myeloid depletion	E2-SA	C2-SA (nonselective depletion)	Clodronate liposomes 24 h before and 24 h after I/R, with MSC-exosome versus vehicle treatment; no selective macrophage rescue	Reduced infarct size and inflammatory readouts after exosome treatment; benefit lost or reduced with depletion. A clodronate-only arm was included and did not differ from PBS for infarct size or cardiac function	Systemic clodronate is not macrophage-lineage-selective, although the authors' clodronate-only control shows that depletion did not itself aggravate the injury; the study does not establish cardiac localization, and the miR-182-depleted vesicles were tested only on cultured macrophages, so necessity of endogenous miR-182 cargo for the cardiac effect is untested	Independent MSC-exosome program, distinct from CDC vesicles [65,66]
Cambier 2017 [66]. PMID:28167565	Rat neonatal cardiomyocyte–macrophage coculture (IV)	EV-YF1-primed bone-marrow-derived macrophages added to oxidatively stressed neonatal rat ventricular myocytes, with or without an IL-10-neutralizing antibody	Macrophage-derived IL-10 is required for the cytoprotection conferred by EV-YF1-primed macrophages in coculture	E–	C3-IV	IL-10 neutralization in the cardiomyocyte–macrophage coculture	Cytoprotection of oxidatively stressed cardiomyocytes was lost when IL-10 was neutralized; recombinant IL-10 reproduced the effect	The experiment is in vitro and uses primed macrophages, not the vesicle preparation delivered in vivo, so it does not establish IL-10 necessity for the cardiac effect of the preparation; the upstream vesicle–cargo link is untested by this perturbation	Same program as [14,65]
Cambier 2017 [66]. PMID:28167565	Rat, I/R (SA)	Naked EV-YF1 oligoribonucleotide, 10 μg; intracoronary by left ventricular cavity injection under aortic cross-clamp; 10 min after reperfusion	Directly supplied EV-YF1 is sufficient to raise macrophage IL-10 and confer protection	E2-SA	C3-SA (sufficiency of the supplied fragment only)	Random allocation to EV-YF1, scrambled-sequence control or vehicle; separate in vitro IL-10 neutralization	Infarct mass 24.3 ± 2.9 mg versus 67.4 ± 10.9 (scramble) and 78.3 ± 4.4 mg (vehicle), n = 5–6; CD68+ macrophages and apoptotic cardiomyocytes both reduced (n = 3 per group)	Sufficiency of a synthetic fragment delivered directly does not isolate delivery by the vesicle preparation; the fragment was also modestly cytoprotective without macrophages, so the macrophage route is not the only one available; histological endpoints rest on three animals per group	Same program as [14,65]

Study and identifier	Species/model (context)	Intervention, dose, route, timing	Claim classified	E category	C category	Causal perturbation	Outcome measured	Principal limitation	Independence warning
Cambier 2017 [66]. PMID:28167565	Rat, I/R (SA)	CDC vesicle fraction; intramyocardial	Endogenous vesicular YF1 is required for protection	E2-SA	C0-SA	Not tested: donor-cell/cargo loss-of-function with preserved vesicle preparation was not performed	No necessity result	The authors state that this would require selective depletion of EV-YF1 from the vesicle preparation, which they could not achieve without disrupting the remaining contents	Same program as [14,65]
Zhu 2022 [69]. PMID:36252111	Rat, infarct (SA)	MSC vesicles; intrapericardial; acute	Vesicles act on lymph-node antigen-presenting cells to induce Tregs; a PP2A–Akt–Foxo3 axis is implicated	E2-SA	C2-SA	Treg depletion with anti-CD25 in vivo; pharmacological PP2A inhibition in vivo; Foxo3 knockdown in vitro only, read out as cytokine transcription; cytokine neutralization; antigen-presenting-cell and Treg tracking	Improved repair and function	The operative compartment is a lymph node, not myocardium. Foxo3 was never perturbed in vivo and Akt was measured but never manipulated, so the named axis is not established as necessary for the cardiac outcome. The authors state that the vesicle-taking antigen-presenting subtype is "not fully defined" and that the mechanism of PP2A activation "remains unclear". Initial infarct area was comparable between groups	Single study—no independent replication

Study and identifier	Species/model (context)	Intervention, dose, route, timing	Claim classified	E category	C category	Causal perturbation	Outcome measured	Principal limitation	Independence warning
Lima Correa 2021, Theranostics [72]. PMID:34815807	Mouse, chronic infarct (SA)	hiPSC cardiovascular-progenitor vesicles, 10^{10}; peri-infarct injection 3 weeks after ligation	Functional improvement occurs without detected generation of new cardiomyocytes (lineage-tracing observation)	E2-SA (function)	C–	Two independent reporter systems: MerCreMer/ZEG (n = 15/group) and MADM (n = 6/group)—an unusually direct dual test	No new cardiomyocytes detected; function improved	A statement about this preparation, model and detection threshold. C– because the observation does not test a mechanism. EdU-positive cardiomyocytes were present in both groups and did not differ, and all were reporter-positive, so of pre-existing origin. Capillary density and hypertrophy were unchanged, and function was assessed only 4–6 weeks after injection, so durability is unknown	Companion study to [73]—same program
Lima Correa 2021, Theranostics [72] (second claim). PMID:34815807	Mouse, chronic infarct (SA)	hiPSC cardiovascular-progenitor vesicles, 10^{10}; peri-infarct injection 3 weeks after ligation	The functional benefit depends in part on vesicular miR-133	E2-SA	C2-SA	Co-delivery of an anti-miR-133 antagomir with the vesicle preparation	Partial abrogation of the functional benefit	Loss of function only: the authors did not test a miR-133 mimic, so sufficiency is untested, and the abrogation was partial, so the cargo is contributory rather than dominant. Antagomir co-delivery does not exclude effects on the preparation itself	Same program as [73]

Study and identifier	Species/model (context)	Intervention, dose, route, timing	Claim classified	E category	C category	Causal perturbation	Outcome measured	Principal limitation	Independence warning
Lima Correa 2021, Cardiovasc Res [73]. PMID:32049348	Mouse, acute and chronic infarct; human cells in vitro (SA/IV)	hiPSC cardiovascular-progenitor vesicles; intramyocardial, 3 weeks after infarction (chronic model) or direct cardiac injection (acute)	The vesicles are associated with a reparative immune profile and are of low immunogenicity	E2-SA	C1-SA/IV (association only)	None—no immune-cell depletion and no cargo manipulation	Reduced cardiac Ly6C-high monocytes and circulating IL-1α, TNF-α and IFN-γ (chronic); reduced pro-inflammatory macrophages, Ly6C-high monocytes and neutrophils with raised IL-10 (acute); no allogeneic T-cell or natural killer activation and no immunoglobulin induction on repeated injection	Purely associative for mechanism. The study contains no cargo-dependence experiment; it reports M1/M2 categories, whose limitations are discussed in Section 4.3	Companion study to [72]—same program

Study and identifier	Species/model (context)	Intervention, dose, route, timing	Claim classified	E category	C category	Causal perturbation	Outcome measured	Principal limitation	Independence warning
Emmert 2024 [83]. PMID:37787585	Pig; closed-chest 90 min mid-LAD balloon occlusion with reperfusion (LA)	Human cardiac progenitor-cell small extracellular vesicles; catheter-based intramyocardial (11 mg, ~10 × 10^{11} particles, NOGA-guided) versus intracoronary (22 mg, ~20 × 10^{11} particles) versus vehicle, within 15 min of reperfusion	The randomized groups showed different contrasts against vehicle, but a direct route effect remains unresolved at unequal doses	E2-LA	C0-LA	Randomized allocation to intramyocardial vesicles (n = 6), intracoronary vesicles (n = 6) or vehicle (n = 5); a separate randomized placebo-controlled intracoronary study (n = 6 versus 5) followed to 3 months	At 1 month, infarct volume was lower after intracoronary delivery (13.5 ± 2.5 mL) than vehicle (29.1 ± 10.7 mL; pairwise p = 0.040), whereas intramyocardial delivery did not differ from vehicle (17.6 ± 8.5 mL; p = 0.153); ejection fraction and infarct size did not reach significance across the three arms (p = 0.137 and p = 0.100). At 2–3 days cardiac output and right ventricular ejection fraction were lower in the intramyocardial arm. In the placebo-controlled study, ejection fraction was higher (p = 0.019) and infarct size lower (p = 0.005) at 3 months with reduced fibrosis and increased vessel density	The only randomized large-animal route comparison available, but route and dose are confounded: the intracoronary arm received twice the particle dose. The authors attribute the early intramyocardial disadvantage to needle injury, a procedural harm rather than a property of the route. No immune, uptake or biodistribution endpoint was measured—the authors state that mechanism was beyond the scope—so C0. The product differs from the cardiosphere-derived preparation used in [70]	Independent group; different product line from [70]

Study and identifier	Species/model (context)	Intervention, dose, route, timing	Claim classified	E category	C category	Causal perturbation	Outcome measured	Principal limitation	Independence warning
Sadtler 2016 [79]. PMID:27081073	Mouse, volumetric muscle loss (SA, cross-organ)	ECM biomaterial scaffold; implantation	The pro-regenerative scaffold environment requires CD4 T cells; an IL-4 receptor contribution is shown for macrophage phenotype only	E2-SA	C3-SA, cross-organ	Rag1-deficient hosts with transfer of wild-type or Rictor-deficient CD4 T cells as a rescue arm; MHC-II-deficient hosts; Il4ra-deficient animals assayed for macrophage CD206	Loss of constructive remodelling and of functional muscle recovery in Rag1-deficient hosts, restored by wild-type but not Rictor-deficient CD4 T cells (running recovery 91.1 ± 3.8 versus 60.1 ± 9.7 and 72.3 ± 7.4)	Skeletal muscle, not myocardium; cardiac relevance is C0. Necessity plus rescue holds for CD4 T cells, but Il4ra-deficient animals were assayed only for macrophage CD206, on a different genetic background, and never for functional recovery, so the conjunctive claim is not fully supported. CD206 induction was lost in Rag1-deficient wounds even without a scaffold	Independent group; sets the standard cardiac studies have not met
Wassenaar 2016 [75]; Mesfin 2025 [77]	Rat; ischemia–reperfusion in [77] (35 min occlusion), subacute and chronic; adult female Sprague Dawley in [77] (SA)	Decellularized myocardial matrix hydrogel; intramyocardial; 1 week in [75], 1 or 8 weeks in [77]	The material is associated with altered myeloid, fibroblast, vascular and lymphatic programs	E2-SA	C1-SA	None—transcriptomic and spatial mapping only	Reduced fibrosis, increased vascularity and improved hemodynamics in [75]; transcriptional and spatial changes only in [77], which reported no functional endpoint	No immune necessity test; not separated from mechanical or direct matrix effects; single (female) sex in [77], whose authors state the study "lacks assessment of cardiac function" and that the pathway analyses "should be interpreted with caution". In [75] macrophage counts by immunohistochemistry did not differ despite higher CD68 transcript, and the M1/M2 comparison was "not conclusive"	Same laboratory lineage (Christman program), including [76,94]

Study and identifier	Species/model (context)	Intervention, dose, route, timing	Claim classified	E category	C category	Causal perturbation	Outcome measured	Principal limitation	Independence warning
Rurik 2022 [56]. PMID:34990237	Mouse, angiotensin II/phenylephrine hypertensive fibrosis — not an ischemic infarct (SA)	In vivo generated FAP-directed CAR T cells; intravenous mRNA–LNP	Transient immune redirection is sufficient to remove activated fibroblasts and improve function	E2-SA	C3-SA for sufficiency only	Targeted ablation of activated fibroblasts; no necessity experiment and no irrelevant-cargo targeted control	Reduced fibrosis; restored function without new cardiomyocytes	Not an ischemic infarct model; a concept demonstration. Sufficiency only: the comparators were saline and non-targeted particles, so the contribution of targeting itself is not isolated. Left ventricular mass did not change significantly and perivascular fibrosis persisted	Independent group

Table S2. Human evidence, classified claim by claim.

The descriptive categories are defined in Section 2.2. Immune target engagement means a measured change in an immune parameter after treatment at a mechanistically relevant time; baseline covariates, including high-sensitivity C-reactive protein, are endotype markers and are not target engagement. Randomization can strengthen the E classification but cannot strengthen the C classification for a mechanism that was not measured. Anti-inflammatory pharmacological trials are included as external mechanistic controls, not as regenerative platforms.

Trial and identifier	Platform and population	n	Claim classified	E category	C category	What was measured mechanistically	Conclusion permitted by the data	Independence/caveat
ACCRUE [88]. PMID:25700037	Individual-patient meta-analysis of 12 randomized trials of cell therapy after acute MI	1,252	Cell therapy after acute MI did not improve clinical events, ejection fraction or ventricular volumes	E3-H	C0-H	No immune parameter was measured in the constituent trials. MACCE 14.0% vs 16.3% (HR 0.86, 95% CI 0.63–1.18); ejection fraction mean difference 0.96% (95% CI −0.2 to 2.1)	Absence of demonstrated efficacy across the included trials; a negative efficacy result does not identify the mechanism of failure	Trials heterogeneous in product and protocol
CADUCEUS [84]. PMID:22336189	Autologous cardiosphere-derived cells; recent MI	31 randomized 2:1; 25 analyzed per protocol (17 cells, 8 control); controls received routine care, not placebo	Scar mass decreased and viable mass increased; ejection fraction unchanged	E1-H	C0-H	None	Safety was the prespecified primary endpoint at 6 months; the imaging findings were designated preliminary efficacy endpoints. Scar mass fell 8.4 g and viable mass rose 13.0 g versus control (p = 0.001 and p = 0.01) with ejection fraction unchanged	Same program as [85] and the preclinical work. Two cell batches showed trisomy 8 and one manufacturing run failed
ALLSTAR [85]. PMID:32749459	Allogeneic cardiosphere-derived cells; 4 wk–12 mo post-MI	142 randomized; 134 treated (90 cells, 44 placebo)	Scar size was not reduced at 6 months; the 12-month primary endpoint was never completed	E2-H (randomized but terminated early and underpowered)	C0-H	None reported	Inconclusive rather than definitively negative for the structural endpoint; end-diastolic volume, end-systolic volume and natriuretic peptide were each lower with treatment at 6 months (p = 0.02), which the authors read as disease-modifying bioactivity	Stopped by the sponsor at a prespecified interim analysis (conditional probability of rejecting the primary null 1.1%); the authors state the truncated trial was not powered for the primary or secondary endpoints at 6 months

Trial and identifier	Platform and population	n	Claim classified	E category	C category	What was measured mechanistically	Conclusion permitted by the data	Independence/caveat
BioVAT-HF [17]. PMID:42202318	Allogeneic iPSC-derived engineered heart muscle, epicardial allograft under immunosuppression; heart failure with ejection fraction ≤35%	20 treated; 16 at the maximal tolerated dose; 12 evaluable at the 3-month interim	Interim within-patient structural and symptom-score changes followed engineered-heart-muscle implantation	E1-H (uncontrolled, interim)	C0-H	None—no immune, graft-survival or mediation endpoint in the treated cohort	Least-squares mean increase in target wall thickness 4.5 mm (90% CI 3.7–5.4, $p < 0.001$), ejection fraction +3.9 percentage points (90% CI 0.9–6.8, $p = 0.04$) and KCCQ overall summary score +6.7 points (90% CI 1.0–12.5, $p = 0.06$)	Open-label and uncontrolled, so no comparator-based efficacy inference is possible. Significance was prespecified at $p < 0.10$ with 90% confidence intervals; the symptom-score result was $p = 0.06$. All patients had at least one adverse event (196 events, 57 serious); three patients died, all adjudicated unrelated to the graft. The histological evidence of human remuscularization rests on the single explanted heart reported in [9], not on this cohort
BAMI [89]. PMID:32860406	Intracoronary bone marrow mononuclear cells; acute MI	375 enrolled against a 3,000-patient target	All-cause mortality; not interpretable	E0-H	C0-H	None	The authors state that the trial "does not qualify for assessing the efficacy", that "no group comparisons are possible" and that the data are descriptive	Independent consortium
CHART-1 [90]. PMID:28025189	Cardiopoietic mesenchymal stromal cells; ischemic HF	315 randomized; 271 underwent a procedure (120 cells, 151 sham)	The composite primary endpoint was not met	E3-H	C0-H	None	Neutral trial: the Mann–Whitney primary estimator was 0.54 (95% CI 0.47–0.61, $p = 0.27$). The responsive subgroup was a baseline end-diastolic volume window of 200–370 mL—patients above as well as below that range did not respond—and the analysis was explicitly post hoc and exploratory	Independent consortium. Fourteen catheter-related serious adverse events occurred, including three tamponades and one aortic dissection

Trial and identifier	Platform and population	n	Claim classified	E category	C category	What was measured mechanistically	Conclusion permitted by the data	Independence/caveat
CONCERT-HF [91]. PMID:33811444	Autologous MSC and c-kit+ cardiac cells; ischemic HF	125	The cardiac-cell arms reduced a composite heart-failure event endpoint; quality of life improved with stromal cells; ejection fraction and scar did not change	E2-H (phase 2, exploratory endpoints)	C0-H	None in the parent report	Exploratory and not powered for clinical endpoints, with no adjustment for multiplicity. The significant result was the composite (6.5% versus 28.1%, p = 0.043); heart-failure hospitalization alone did not reach significance (3.2% versus 21.9%, p = 0.053). The authors state the findings are "not conclusive but, rather, hypothesis generating"	Parent trial of [95]
CONCERT-HF biomarker analysis [95]	Prespecified secondary plasma analysis of the CONCERT-HF population	96 of 125 (samples unavailable for 29)	Circulating inflammatory and matrix-remodelling proteins show selective, time-dependent treatment-associated changes	E–	C1-H	Plasma TNF-α, PTX3, PDGF-BB, GDF-15, BMP-9 and MMP-1 at baseline and days 7, 30 and 180	Circulating target engagement is demonstrated, but narrowly: after false-discovery-rate correction only pentraxin-3 and PDGF-BB survived, and only PDGF-BB correlated with a clinical outcome	Same program as [91]; denominator differs from the parent trial
CONCERT-HF biomarker analysis [95]	Prespecified secondary plasma analysis of the CONCERT-HF population	96 of 125 (samples unavailable for 29)	The circulating biomarker changes localize to the myocardium	E–	C0-H	No myocardial biomarker or tissue measurement	Cardiac localization is not supported	Same program as [91]; denominator differs from the parent trial
CONCERT-HF biomarker analysis [95]	Prespecified secondary plasma analysis of the CONCERT-HF population	96 of 125 (samples unavailable for 29)	The circulating biomarker changes mediate the exploratory clinical effects	E–	C0-H	No mediation analysis	Mediation is not supported	Same program as [91]; denominator differs from the parent trial

Trial and identifier	Platform and population	n	Claim classified	E category	C category	What was measured mechanistically	Conclusion permitted by the data	Independence/caveat
DREAM-HF [13]. PMID:36858705	Allogeneic mesenchymal precursor cells; HFrEF	565	Time to recurrent decompensated HF events or resuscitated ventricular arrhythmias was not improved; MI or stroke was reduced in a prespecified analysis	E3-H (primary endpoint, HR 1.17, 0.81–1.69); E2-H (prespecified MI-or-stroke composite in the 537-patient analysis population, HR 0.42, 0.23–0.76); E1-H (post hoc 3-point MACE)	C0-H	Baseline hsCRP only, with the ≥2 mg/L threshold and the subgroup analysis both prespecified (threshold carried over from CANTOS); no on-treatment engagement measure	Neutral primary endpoint. Ejection fraction rose modestly at 12 months ($p = 0.041$), confined to the elevated-hsCRP subgroup. The inflammation-related findings motivate prospective enrichment, not a mechanistic conclusion	Independent sponsor
SCIENCE [93]. PMID:36644821	Allogeneic adipose-derived stromal cells; chronic ischemic HFrEF	133	No improvement in end-systolic volume, ejection fraction, symptoms, walk distance, NT-proBNP or CRP	E3-H	C1-H (CRP measured and unchanged—a negative engagement result)	Systemic CRP	Neutral trial with an explicitly negative inflammatory readout. The primary endpoint, change in end-systolic volume at 6 months, was 0.3 ± 5.0 mL ($p = 0.945$); ejection fraction numerically favoured placebo (−1.6 ± 1.0%, $p = 0.119$). Safety was maintained over 3 years	Independent European consortium
POSEIDON [92]. PMID:23117550	Allogeneic versus autologous MSC; ischemic cardiomyopathy	31 randomized, 30 treated	Chamber geometry changed; ejection fraction did not improve	E1-H	C0-H	Limited immunologic safety assessment only	Safety and dose comparison. Ejection fraction did not improve (+1.96, 95% CI −0.47 to 4.39, $p = 0.11$); the infarct-size reduction was measured by computed tomography rather than magnetic resonance, and the lower 20-million-cell dose outperformed the 200-million dose	Independent group; open-label with no placebo arm, which the authors name as the principal limitation. Two allogeneic recipients developed de novo HLA class I antibodies

Trial and identifier	Platform and population	n	Claim classified	E category	C category	What was measured mechanistically	Conclusion permitted by the data	Independence/caveat
ESCORT [20]. PMID:29389360	hESC-derived cardiovascular progenitors in a fibrin patch; severe ischemic LV dysfunction	6 (median dose 8.2 × 10^6 cells; 1 early death, 1 death at 22 mo)	Delivery is feasible and short-term safety was acceptable; engraftment was not demonstrated (direct observation)	E0-H	C–	Donor-specific antibodies; circulating donor-derived DNA; autopsy in the patient who died on day 9	Feasibility and short-term safety only. At autopsy the patch was identified with no noticeable inflammatory response, and 3 of 6 patients developed clinically silent alloimmunization	The patch was delivered during coronary bypass surgery; the authors state that the small sample, absence of blinded assessment and the concomitant grafting preclude any conclusion on efficacy, and they attribute any contribution of the cells to secretion rather than to contractile replacement. Wall motion improved in non-revascularized cell-treated segments (3 of the 4 patients contributing 1-year data), which mitigates but does not remove the confound
VentriGel [94]. PMID:31709316	Decellularized myocardial matrix hydrogel; 60 d–3 y post-MI	15	Delivery is feasible; NYHA class and 6-minute walk distance improved	E1-H (uncontrolled)	C0-H	None	No mechanistic or efficacy inference is possible. Walk distance improved at 3 and 6 months (+35.6 m, $p = 0.033$; +44.4 m, $p = 0.007$), whereas the NYHA and quality-of-life improvements reached significance only at one month	Same laboratory lineage as [75,77,76]
AUGMENT-HF [86]. PMID:26082085	Alginate hydrogel, mechanical rationale; advanced HF	78	Peak VO_2 improved versus standard therapy	E2-H	C0-H (immune mediation not tested and not intended)	None	Functional benefit can occur with no immune claim, and this trial is retained as the nonimmune comparator. Peak oxygen uptake rose 1.24 mL/kg/min (95% CI 0.26–2.23, $p = 0.014$)	Independent sponsor. Unblinded by design, since sham thoracotomy was judged to be unacceptable. Thirty-day mortality was 8.6% in the treated arm versus none in control, with a 25% major surgical complication rate—a harm signal that must accompany any citation of the functional result

Trial and identifier	Platform and population	n	Claim classified	E category	C category	What was measured mechanistically	Conclusion permitted by the data	Independence/caveat
CANTOS [96]. PMID:28845751	Canakinumab (anti-IL-1β); prior MI, hsCRP ≥2 mg/L	10,061	Recurrent major cardiovascular events were reduced at the 150 mg dose only	E3-H (150 mg dose)	C–	Randomized clinical outcome comparison	Only the 150 mg dose met the prespecified multiplicity-adjusted threshold (HR 0.85, 0.74–0.98); the 300 mg dose did not. All-cause mortality was unchanged (HR 0.94, 0.83–1.06) and fatal infection was more frequent than with placebo. This is not evidence of repair or regeneration	External mechanistic control, not a regenerative platform
CANTOS [96]. PMID:28845751	Canakinumab (anti-IL-1β); prior MI, hsCRP ≥2 mg/L	10,061	Systemic inflammatory target engagement occurred	E–	C1-H	On-treatment hsCRP reduction (median reduction ~37 percentage points greater than placebo at 150 mg)	Systemic target engagement was demonstrated	External mechanistic control, not a regenerative platform
CANTOS [96]. PMID:28845751	Canakinumab (anti-IL-1β); prior MI, hsCRP ≥2 mg/L	10,061	Systemic target engagement mediated the clinical benefit	E–	C0-H	No mediation analysis	Mediation was not established	External mechanistic control, not a regenerative platform
ASSAIL-MI [97]. PMID:33858620	Tocilizumab (anti-IL-6R); STEMI at primary PCI	199	Claim 1—structural treatment effect: the myocardial salvage index increased and microvascular obstruction decreased	E3-H (randomized CMR endpoint as tested)	C–	Cardiac magnetic resonance at 3–7 days; 101 tocilizumab vs 98 placebo; salvage index difference 5.6 percentage points (95% CI 0.2–11.3, $p = 0.04$); microvascular obstruction less extensive ($p = 0.03$)	A randomized structural benefit limited to the endpoint tested. Final infarct size (7.2% vs 9.1%, $p = 0.08$), baseline-adjusted left ventricular volume ($p = 0.54$) and 6-month NT-proBNP ($p = 0.25$) did not differ	External mechanistic control, not a regenerative platform
ASSAIL-MI [97] (claim 2). PMID:33858620	As above	199	Claim 2—systemic inflammatory target engagement occurred	E–	C1-H	C-reactive protein area under the curve during hospitalization, substantially lower with tocilizumab ($p < 0.001$)	Target engagement is robustly demonstrated at the systemic level	External mechanistic control
ASSAIL-MI [97] (claim 3). PMID:33858620	As above	199	Claim 3—the structural benefit was causally mediated by inflammatory target engagement	E–	C0-H	None; no mediation analysis was performed	Not supported. Randomization to treatment cannot demonstrate that a biomarker change lay on the causal path	External mechanistic control
ASSAIL-MI [97] (claim 4). PMID:33858620	As above	199	Claim 4—cardiac cellular localization or myocardial immune reprogramming occurred	E–	C0-H	Neither was measured	Not supported by any measurement in this trial	External mechanistic control

Trial and identifier	Platform and population	n	Claim classified	E category	C category	What was measured mechanistically	Conclusion permitted by the data	Independence/caveat
VCU-ART3 [87]. PMID:32122219	Anakinra (IL-1 receptor antagonist); STEMI	99	The randomized intervention reduced the systemic inflammatory response	E3-H (inflammatory primary endpoint)	C1-H	C-reactive protein area under the curve: 67 (IQR 39–120) versus 214 (IQR 131–394) mg·day/L, $p < 0.001$	Systemic inflammatory target engagement was demonstrated	External mechanistic control
VCU-ART3 [87]. PMID:32122219	Anakinra (IL-1 receptor antagonist); STEMI	99	The randomized intervention changed ventricular volumes or ejection fraction	E3-H	C0-H	Echocardiography. Infarct size was not an endpoint: no magnetic resonance was performed, and the enzymatic estimate appears only as a baseline characteristic	Neither end-systolic volume ($p = 0.21$) nor ejection fraction ($p = 0.61$) changed. The trial does not speak to infarct size in either direction	External mechanistic control. Only 77 of 99 had complete biomarker data and 63 of 99 had paired echocardiograms
APEX-AMI [53]. PMID:17200474	Pexelizumab (anti-C5); STEMI at primary PCI	5,745	Mortality was not reduced	E3-H	C1-H (target engagement was achieved)	Hemolytic complement activity in a 229-patient pharmacodynamic subset: 90% of treated patients achieved >70% inhibition at baseline and 24 h	Suppression of the pathway was achieved and outcome was still unchanged, so incomplete engagement does not explain the null result	Enrolment was reduced from a planned 8,500 to 5,745, leaving ~52% power for the anticipated 24% mortality reduction; observed HR 1.04 (0.80–1.35)
COVERT-MI [98]	High-dose colchicine at reperfusion; STEMI	195 randomized, 192 analyzed	CMR infarct size was not reduced at 5 days or 3 months	E3-H	C0-H (no myocardial engagement demonstrated)	None at the myocardial level	Broad anti-inflammatory treatment is not equivalent to immune-guided repair. Infarct size was 26.0 versus 28.4 g ($p = 0.87$) at 5 days and unchanged at 3 months	External mechanistic control. Left ventricular thrombus was more frequent with colchicine (22.2% versus 7.4%, $p = 0.01$) and, for the largest areas at risk, treatment was associated with a larger resulting infarct (interaction $p = 0.03$)—direct evidence that untargeted immune suppression can harm

Footnote to Tables S1 and S2: E– and C– indicate that the corresponding axis is not applicable to the claim, not that the evidence is weak; directness and limitations are reported in the perturbation and limitation columns. Denominators are reported per endpoint when they differ from the randomized population (e.g., 96 of 125 randomized participants in the CONCERT-HF biomarker analysis). Independence warnings identify shared laboratories, product lines or experimental programs and therefore non-independent evidence.

Abbreviations used in Tables S1 and S2: BAK, BCL2 antagonist/killer 1; BAX, BCL2-associated X protein; BMP-9, bone morphogenetic protein 9; CAR, chimeric antigen receptor; CCR2, C-C chemokine receptor type 2; CD, cluster of differentiation (CD4, CD14, CD25, CD31, CD45, CD68, CD163, CD206); CDC, cardiosphere-derived cell; CI, confidence interval; CMR, cardiovascular magnetic resonance; COMP, cartilage oligomeric matrix protein (also known as thrombospondin-5); CRP, C-reactive protein; DTR, diphtheria toxin receptor; ECM, extracellular matrix; EHM, engineered heart muscle; EV, extracellular vesicle; GDF-15, growth differentiation factor 15; GFP, green fluorescent protein; GvHD, graft-versus-host disease; HF, heart failure; HFrEF, heart failure with reduced ejection fraction; HLA, human leukocyte antigen; HR, hazard ratio; IDO, indoleamine 2,3-dioxygenase; IFN, interferon; IL, interleukin; IQR, interquartile range; IRF3, interferon regulatory factor 3; KCCQ, Kansas City Cardiomyopathy Questionnaire; LAD, left anterior descending coronary artery; LNP, lipid nanoparticle; LV, left ventricular; LVEF, left ventricular ejection fraction; Ly6C, lymphocyte antigen 6 complex locus C; MACCE, major adverse cardiac and cerebrovascular events; MACE, major adverse cardiovascular events; MADM, mosaic analysis with double markers; MERFISH, multiplexed error-robust fluorescence in situ hybridization; MerTK, MER proto-oncogene tyrosine kinase; MHC, major histocompatibility complex; MI, myocardial infarction; MISEV2023, Minimal Information for Studies of Extracellular Vesicles 2023; MMP, matrix metalloproteinase; MSC, mesenchymal stromal cell; NGAL, neutrophil gelatinase-associated lipocalin; NOD/SCID, nonobese diabetic/severe combined immunodeficiency; NT-proBNP, N-terminal pro-B-type natriuretic peptide; NYHA, New York Heart Association; PCI, percutaneous coronary intervention; PDGF-BB, platelet-derived growth factor BB; PP2A, protein phosphatase 2A; PTX3, pentraxin-3; STEMI, ST-elevation myocardial infarction; STING, stimulator of interferon genes; TCR, T-cell

receptor; TIMD4, T-cell immunoglobulin and mucin domain containing 4; TNF, tumour necrosis factor; TREX1, three-prime repair exonuclease 1; TSG-6, TNF-α-stimulated gene 6 protein. The evidence codes E–, E0–E3, C–, C0–C3 and the context tags H, LA, SA and IV are defined in Section 2.2 of the main manuscript.
